# One- and Five-Ringgit Malaysia banknotes reader with counterfeit detection for visually impaired person using backlight mechanism and image processing techniques

**DOI:** 10.12688/f1000research.58446.2

**Published:** 2022-04-13

**Authors:** Turki Khaled Salem, Wai Kit Wong, Thu Soe Min, Eng Kiong Wong

**Affiliations:** 1 Faculty of Engineering and Technology, Multimedia University, BKT Beruang, Melaka, 75450, Malaysia

**Keywords:** Circuit and System, Banknote Reader, Image Processing, Banknote Counterfeit, Ringgit Detector

## Abstract

Visually impaired persons face challenges in running business activities, especially in handling banknotes. Malaysia researchers had proposed some Ringgit banknotes recognition systems to aid visually impaired persons recognize and classify Ringgit banknotes. However, these electronic banknote readers can only recognize Malaysian Banknotes’ Ringgit value, they have no counterfeit detection features. The purpose of this study is to develop a banknote reader that not only can help visually impaired persons recognize the banknote value, but also to detect the counterfeit of the banknote, safeguarding their losses. This paper proposed a Malaysian banknote reader using backlight mechanism and image processing techniques to read and detect counterfeit for one Ringgit and five Ringgit Malaysian banknotes. The developed handheld banknote reader used visual type sensor to capture banknote image, passed to raspberry pi controller to perform image processing on banknote value and the extracted watermarks features. The developed image processing algorithm will trace out the region of interests: 1)see-thru windows, 2)Crescent and Star, 3)Perfect see though register and detect the watermarks features accordingly. The processed result will be passed back to the handheld banknote reader and broadcast on an attached mini speaker to aid the visually impaired understand the holding banknote, whether it is a real one Ringgit, real five Ringgit or none of them. The experimental result shown by this approach able to accomplish numerous round of banknote reading attempts with successful outcomes. Confusion matrix is further employed to study the performance of the banknote reader, in terms of true positive, true negative, false positive and false negative. Details analysis had been focused on the critical false positive cases (predicted real banknote and actually is fake banknote) and false negative cases (predicted fake banknote and it is actually real banknote).

## 1. Introduction

Banknote readers are machines that are used to check whether the received banknotes are genuine or fake. These devices can be found in a variety of automated equipment, including supermarket self-check-out machines, laundromat washing machines, parking ticket paying machines, automatic fare collecting machines, public transportation ticket selling machines, and vending machines. The operating procedures for these machines’ banknotes reading devices entail inspecting the banknotes that have been entered into the machine and running a series of tests to see if they are counterfeit or not. These currency acceptors must be accurately configured for each item to be accepted since the specifications for each banknote are different.

Generally, the banknote reader suitable for Malaysian banknotes can be categorized into four processes: FEEL, LOOK, TILT and CHECK. ^1^ Feel is defined by the banknote substrate’s quality. Polymer banknotes featuring raised print effect on the picture of the first Seri Paduka Baginda Yang di-Pertuan Agong and words made of special plastic. Look involves examining the banknote under the light of a white bulb. A three-dimensional watermark portrait will appear, as well as a perfect see-through registration and a clear window. The security thread will appear in a continuous dark-colored line. Tilt involves tilting the banknote while holding it straight. Examine means examining the security thread and the coloured glossy patch for image and colour changes. Simple equipment may be used to check the banknote, except for certain security features, the Ultraviolet light device will not cause the paper substrate to glow. Micro-letterings will be easily apparent with a magnifying lens. By using the “FEEL, LOOK, TILT, and CHECK” principle, all current Malaysian banknotes counterfeits can be identified clearly without much trouble.

**Figure 1.**
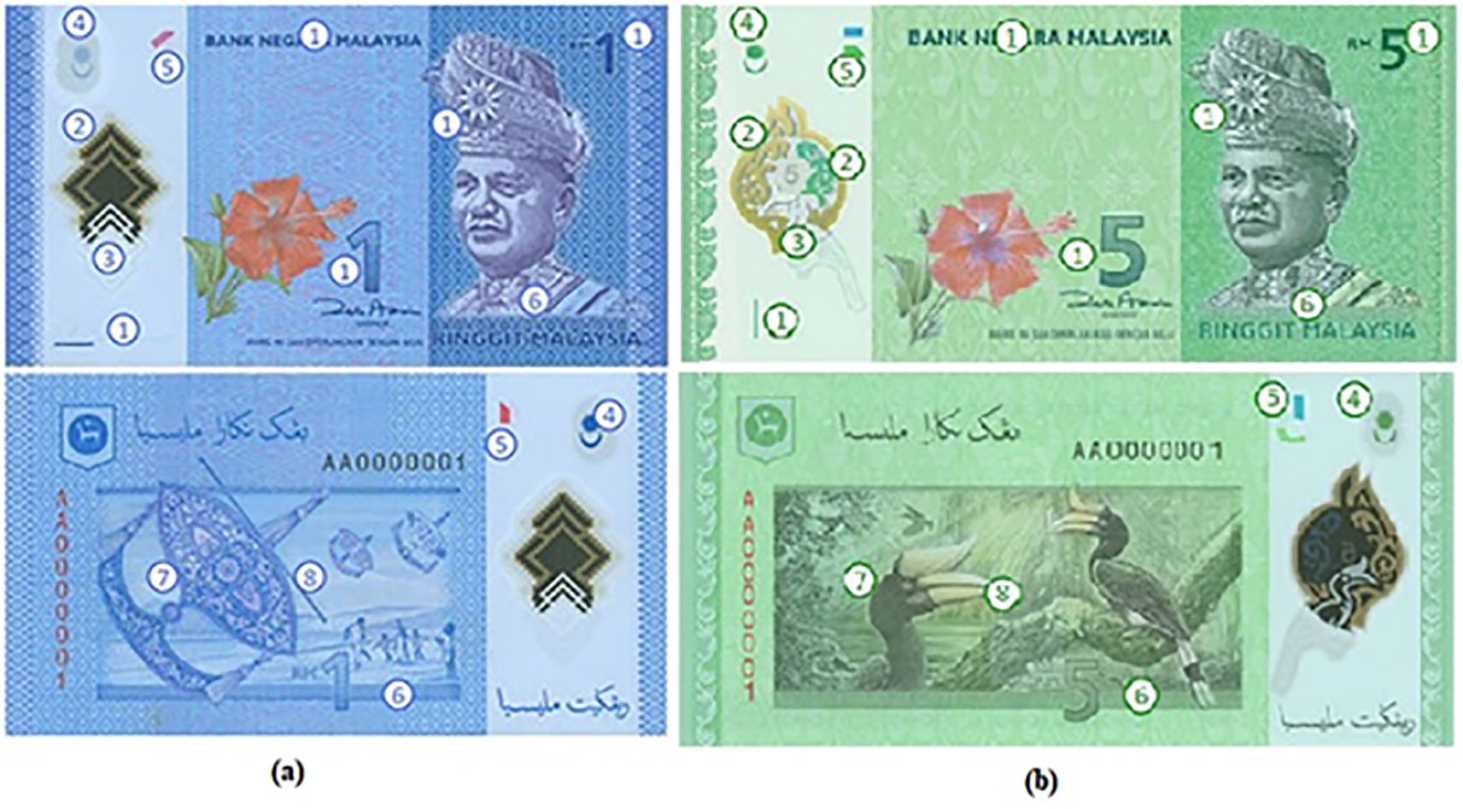
(a) RM1 and (b) RM5 banknotes and their corresponding watermarks.

The RM1 and RM5 Malaysian Banknotes are shown in Figure 1a and Figure 1b respectively. These banknotes are made from polymer substrate and with security features/watermarks with label 1-8. In sequence: 1) Intaglo, 2) Clear Window, 3) Shadow Image, 4) Crescent & Star Non-transparent window, 5) Perfect see thru Register, 6) Micro-Lettering, 7) Two color fluorescent element for Perfect see-thru, 8) UV BNM Text and Logo. Among the eight types of watermarks, there are three types of watermarks that related to the use of front-backlight mechanism 1) see-thru windows, 2) Crescent and Star non transparent window, 3) Perfect see though register). These three types of watermarks will be selected for the proposed prototype to run test.

A person who is visually impaired has a vision problem that may not be corrected by wearing glasses. The difference between a blind person and visually impaired person is that the impaired is dim-sighted or visually challenged, not entirely blind, whereas the blind person is entirely blind. ^2^ The challenges experienced by the visually impaired people at conducting daily-life activities, particularly in operating a business, shopping and tasks involving banknotes handling, are similar to those experienced by blind people. A visually impaired person’s banknote transaction is usually handled by their accompanying trusted business vendor or a partner. However, this scenario puts the visually impaired person in danger of being duped in restricting the commercial activities by the accompanying partner or trusted business vendor.

The Bulgarian Cash Vision team developed the ‘b-note system’, ^3^ a banknote scanner that helps visually handicapped Bulgarians recognize Bulgarian money. They developed a tiny box scanner that employs the camera sensor of a Raspberry Pi controller to record the bill’s middle section of an image using feature extraction algorithm to detect the minimal value (specific stamped marks at one of the banknote’s corners) and the value of the banknote currency. This banknote reader is not suitable to detect Malaysian banknotes because there are no engraved indications on Malaysian banknotes.

NantMobile Money Reader, developed by IPPLEX, ^4^ allows users to aim their iOS device’s camera at a banknote and receive real-time denomination information. It accepts 21 different countries’ currencies, covering the US dollar, Singapore dollar, Australian dollar, etc. The Malaysian ringgit is also disclosed in the reader’s directory. However, this product is just an application software that allowed users to download and install physically on devices such as an iPhone, iPad, or smart tablet to use. The use of a touchscreen is inconvenient for blind and visually impaired people.

Convolutional Neural Networks using MobileNet model was selected by Ref. 5 in detecting Ethiopian banknotes. Convolutional Neural Networks using Canny Edge detection and multiscale template matching methods were selected by Ref. 6 in detecting Indian banknotes. Both these two models are detecting banknotes denomination and counterfeit. However, their counterfeit detection only focus on banknotes’ surface security features, like micro-lettering and only can detect single - sided of banknotes. Unlike other hidden type of watermarks, micro-lettering is easy to be printed by current high-resolution printers.

To assess Malaysian banknotes denomination, Universiti Teknologi Malaysia’s researchers presented a banknote recognizer with sensor-based modality. ^7^ The system employs an Arduino UNO as the processing component, which has a hefty physical architecture that makes it impractical for holding by consumers. Aside from that, the rule-based technique to identify the worth banknotes is intuitively established, with no classifier intervention or machine learning in the banknote interpretation. In 2018, the same researchers used Arduino Lilypad to improve the recognizer of banknote into a wearable device for identify the Malaysian Ringgit banknote. ^8^ The TCS 34725 colour sensor data was fed into a suggested embedded decision tree classifier, which was then tested using 10-folder cross validation and compared to the k-Nearest Neighbour (k-NN) and Nave Bayesian classifiers.

The disadvantage of the Malaysian banknote readers proposed above are no counterfeit detection. The huge size in device will make it difficult to carry by visually impaired person. Therefore, the proposed Malaysian Banknote reader in this paper will relook into the embedded system design to solve the problem of the bulky size reader. Other than that, counterfeit detection will be embedded into the proposed Malaysian Banknote reader to detect the counterfeit of the banknote, safeguarding the users’ losses.

In this paper, a vision based Malaysian banknote reader has been designed to handle Malaysian banknotes for visually impaired people in order to improve the present Malaysian banknote reader and to meet the needs of visually impaired people when doing their regular business operations.

Different values of Malaysian banknotes are having different types of watermarks, for examples RM1 and RM5 required backlight mechanism, Tilting/rotating mechanisms were necessary for the RM10 and RM20, while ultraviolet light shooting mechanisms were necessary for the RM50 and RM100. The current developed banknote reader work is focused on recognized RM1 and RM5, with backlight mechanism and corresponding image recognition techniques.

The proposed Malaysian banknotes reader’s hardware components include a microprocessor for camera control, a speaker module and illumination. The primary operating idea is that the image of the banknote is captured by a camera, transmitted to the microcontroller for image processing. The developed image processing algorithm will trace out the region of interests: 1) see-thru windows, 2) Crescent and Star, 3) Perfect see though register, from the captured images and detect the watermarks features accordingly to decide the values and counterfeit for the inserted banknote. The detection results are then played as voice message on a mini speaker embedded on the banknote reader. This banknote detection system has a success rate of up to 89% in identifying the proper banknote value and counterfeit.

The research contribution and novelty for this work is a new model of Malaysian banknotes counterfeit detection using watermarks image processing analysis and classifier with fuzzy logic. In particular, the three watermarks features: 1) see-thru windows, 2) Crescent and Star, 3) Perfect see though register will be extracted from the one Ringgit and five Ringgit banknotes to determine the real/fake in a dynamic environment with ambiguous, distorted or imprecise banknotes images.

The paper is well ordered in following manner. The Malaysian banknote reader system model with backlight mechanism will be briefly detailed in Section 2. Section 3 show the proposed image processing-based RM1 and RM5 Malaysian banknotes detection algorithm. Section 4 comments same experimental result and lastly in Section 5, conclusion is future work are presented.

## 2. RM1 and RM5 banknotes reader system model

**Figure 2.**
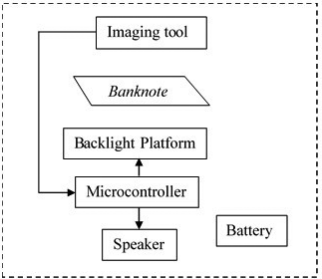
RM1 and RM5 banknotes detection system model.

The system model for the RM1 and RM5 banknotes reading system is show in Figure 2. The banknotes detector is consisting of various parts and a slot of banknote insertion. The working principle start with the backlight platform with white light is turned on/off to captured two images of the inserted banknote, one with backlight and one with no backlight images. The two captured images are sent to microcontroller for image processing and check if the inserted banknote is a real RM1, real RM 5 or fake banknote/none of them. The results will be displayed on a speaker to allow the visually impaired person knows the holding paper notes.

### 2.1 Micro-controller

The micro-controller is used to regulate the functionalities of embedded systems in the banknotes detection system. Two types of micro-controllers surveyed. In Type1, an Arduino was surveyed. The CPU, RAM, and ROM are all found on the Arduino board’s Micro-controller. All of the extra hardware on the Arduino Board is used for power, programming, and IO connectivity. In Type2, Raspberry Pi 4 Model B was surveyed. Raspberry Pi 4 Model B is a single-board computer, with CPU, memory, and graphics chip soldered together on a single circuit board. The Arduino clock speed is 16 MHz, while the Raspberry Pi clock speed is roughly 1.2 GHz. Raspberry Pi is ideal for writing Python-based software, but Arduino is ideal for connecting sensors and controlling LEDs and motors. The Raspberry Pi includes Bluetooth and Wi-Fi technology on board, whereas the Arduino does not have wireless connectivity. Raspberry Pi can simply connect to the internet via Wi-Fi, whereas the Arduino requires an extra module to do so. Therefore, taking into consideration of the above advantages, type 2 micro-controller, the Raspberry Pi 4 Model B is selected to be used in this project.

### 2.2 Imaging tool

An appropriate imaging tool capable of taking a perfect image of the banknote is selected, allowing the image to be processed accurately. Three types of imaging tool are surveyed. In Type 1, a Raspberry Pi 5MP camera sensor board was surveyed. The sensor itself features a fixed focus lens and a native resolution of 5 megapixels. It can capture static photos with a resolution of 2592 by 1944 pixels. In Type 2, a 5MP OV5647 Fisheye Camera Module for Raspberry Pi was surveyed. This imaging set improves optical performance and provides a clearer, sharper image as well as an integrated IR filter. However, the static photos only have a resolution of 2592 × 1944 pixels. In Type 3, a Raspberry Pi 8MP Camera Module V2 was surveyed. The Raspberry Pi Camera Module V2 is the Raspberry Pi Foundation’s new upgraded official camera board, with an ultra-high-quality 8MP (megapixel) sensor and a fixed focus lens. This V2 camera module can capture static photos at a resolution of 3280 × 2464 pixels. Type 3 imaging tool is selected to be used in this project due to the better resolution and finer focus range.

### 2.3 Backlight platform

The purpose of having a backlight platform is to illuminate the banknotes from the back to aid the imaging tool captured the watermarks (see-thru windows, Crescent and Star, Perfect see though register) hidden in the real RM1 and RM5 banknotes. A custom-made therapy LED white Light with 3 dimming levels and USB powered cable had been fabricated. The maximum light intensity generated is 12000LUX and with the box size of dimension 235 mm (L) × 142 mm (W) × 16 mm (H), fit with the Malaysian RM1 and RM5 banknotes sizes.

### 2.4 Speaker

**Figure 3.**
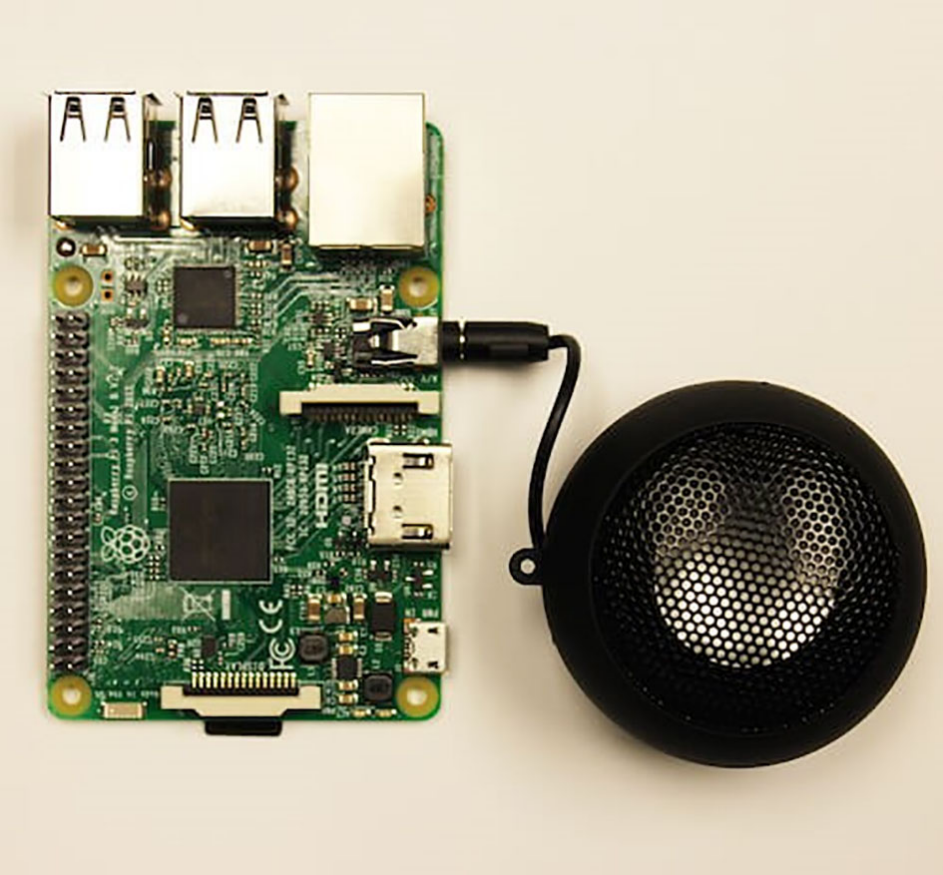
Mini speaker.

The speaker module is applied to output the voice message of the banknote values to the visually impaired person. This is because the visually impaired individual can only “hear” but not “see” the output. As such, the Mini speaker module as shown in Figure 3 is chosen. The module can be controlling using Raspberry Pi. Using a software interface, the Raspberry Pi can convert text to speech and played it on the mini speaker module. The mini speaker module has a very compact size of 5 cm × 3.5 cm (Diameter × Height), which is quite appealing because the system’s hardware should be as tiny as feasible. The notification messages given to the users include: “Real one Ringgit”, “Real five Ringgit” and “Not a Malaysian banknote.”

The description on how to set up this sound/notification is given below: Import pyttsx3 library in Python. It is a text-to-speech conversion library in Python. The results in step 6 Decision making part will be sent to activate the text (e.g. Real one Ringgit, Real five Ringgit or Not a Malaysian banknote.” The pyttsx3 command will transfer the text to speech and display at the speaker. Below is the sample of codings:

engine = pyttsx3.init()

engine.say("Real five ringgit")

engine.runAndWait()

engine.stop()

### 2.5 Battery

The entire system consumed up to current rating of 1.2 A and voltage rating of 5.0 V. A power bank with a 5 V output can be selected as a power source for this project. The power bank is the power source to Raspberry Pi using Type-C connectors. Raspberry Pi will supply direct power to the speaker module and imaging tools. The purpose of employing a power bank as a power source rather than a power line or socket is to produce a portable gadget that can be carried about. Furthermore, the size of the handheld banknote reader should be as compact as feasible, and cumbersome power sources should be avoided.

**Figure 4.**
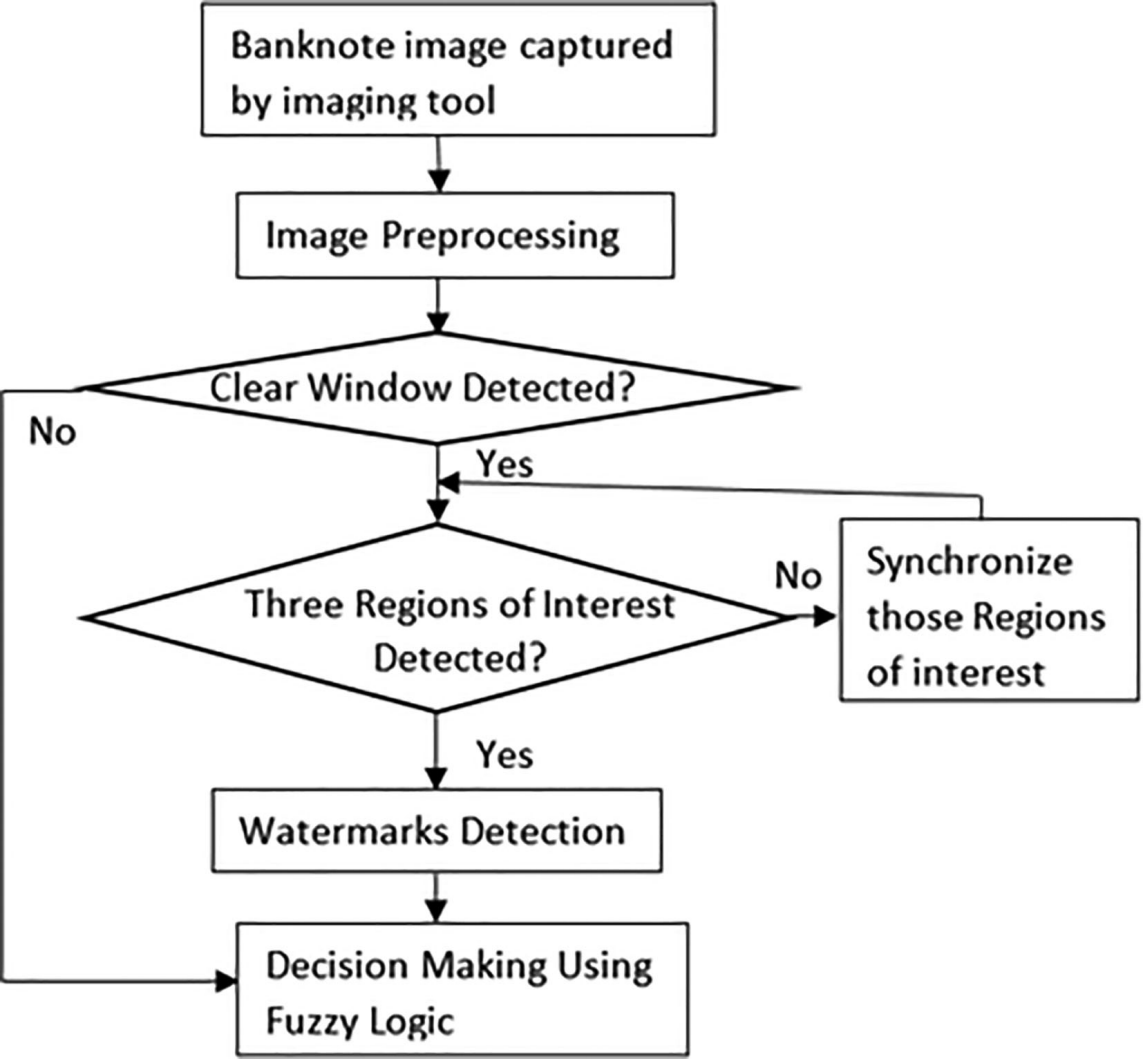
Methodology of the proposed image processing model.

Figure 4 shown the methodology proposed by the authors to solve the problem statement of banknote counterfeit detection. The detail of the algorithm is explained in Section 3.

## 3. RM1 and RM5 banknotes detection image processing algorithm

The image processing algorithm for RM1 and RM5 detection can be divided into 6 steps:

Step 1: Banknote image acquisition

**Figure 5.**
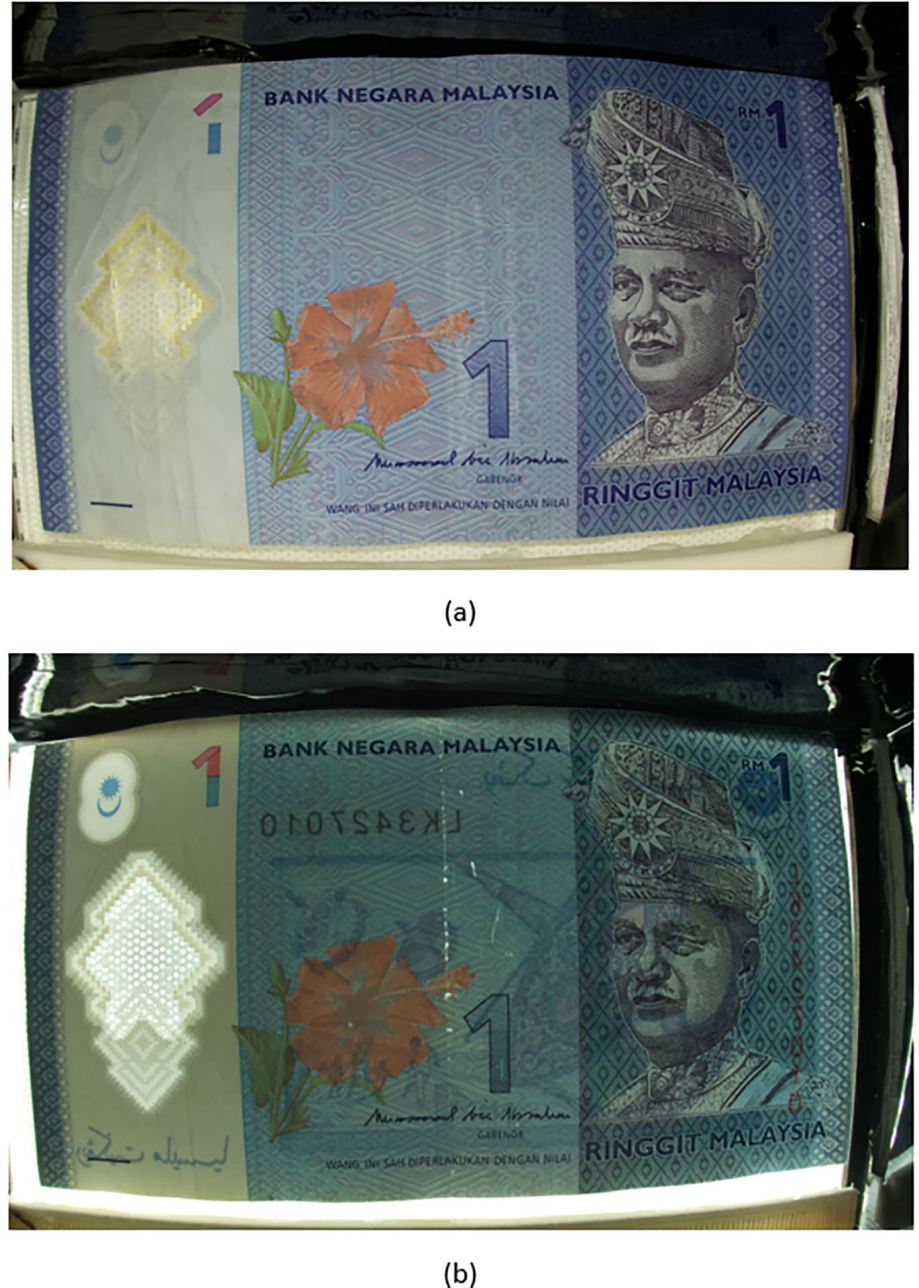
Sample set of acquired RM1 banknote (a) image “Ba”, (b) image “Bb”.

Turn off the back lamp, imaging tool takes image of the slotted in banknote and save it as image “Ba”. Turn on the back lamp, imaging tool take image of the slotted in banknote and save it as image “Bb”. A sample set of the RM1 banknote (image “Ba” and “Bb”) is shown in Figure 5 below.

Step 2: Image pre-processing

Improve the image quality and reduce image noise by converting image “Bb” from RGB colour to grey scale colour. ^9^

The two sub-steps below applied for image preprocessing:

**1.** Resize image

Certain images capture by the imaging tool and pass to the image processing tasks are in different sizes, these images should be standardized in size. Resize all input images (Ba and Bb) to standard size images using the below equation:

$$\left[\mathrm{Ba},\mathrm{Bb}\right]=\text{Resized}\;\left(\text{width, height, no. RGBchannels}\right)$$ (1)

**2.** Remove image noise

**Figure 6.**
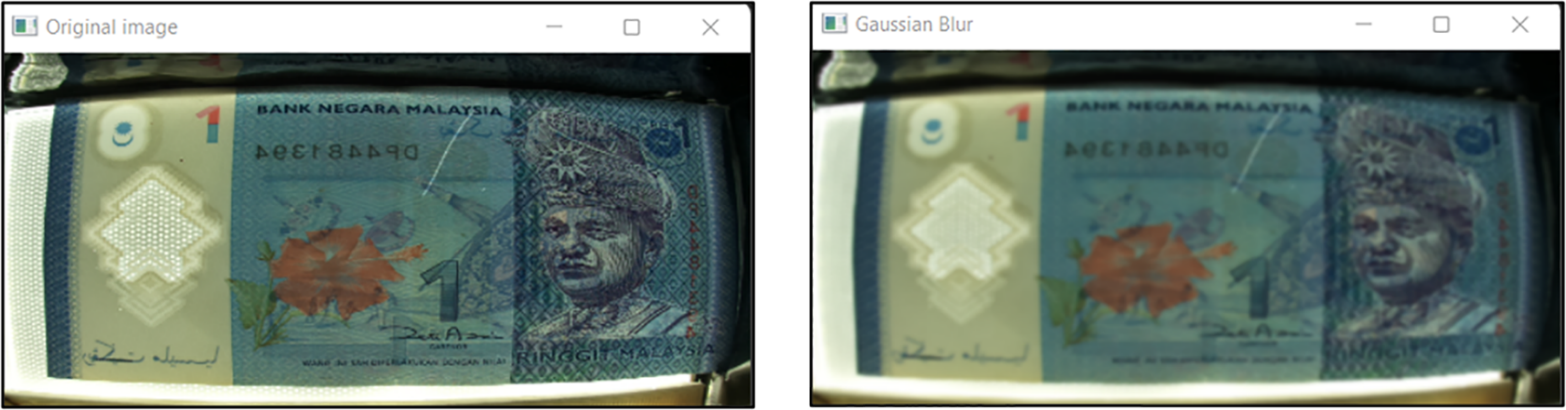
Original and Gaussian Blur converted image.

Using Gaussian Blur function image Processing method ^10^ to remove the unwanted noise on images “Ba” and “Bb”. A sample image “Ba” of RM1 is shown in Figure 6, on the original image and the Gaussian Blur converted image.

Step 3: Songket/Hornbill clear window detection

**Figure 7.**
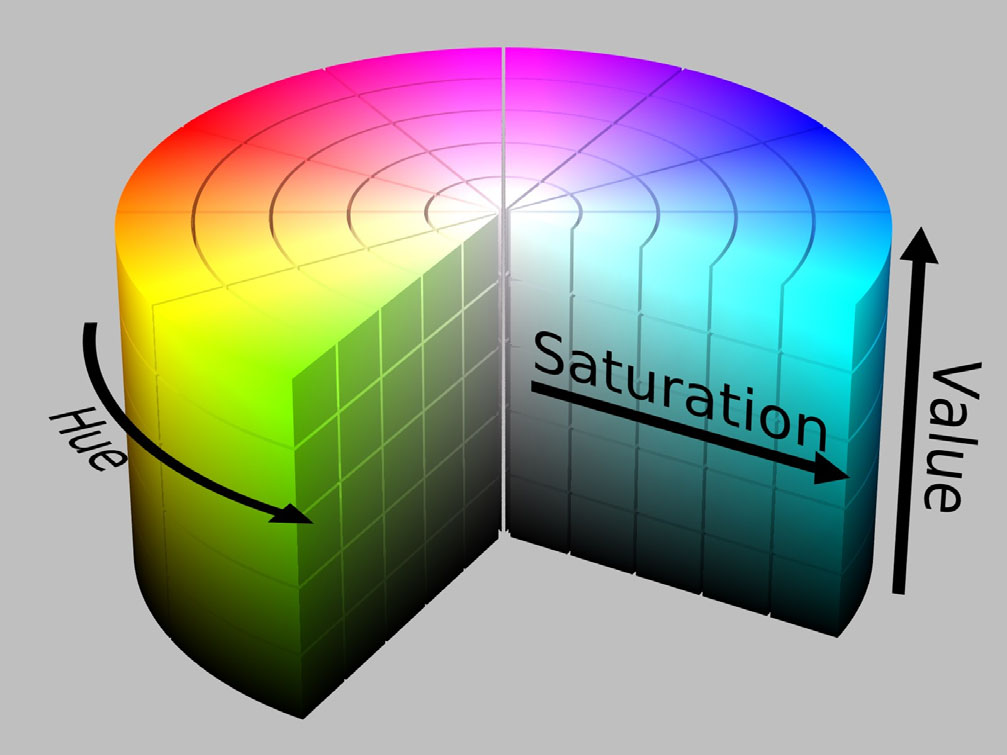
HSV colour model. ^12^

**Figure 8.**
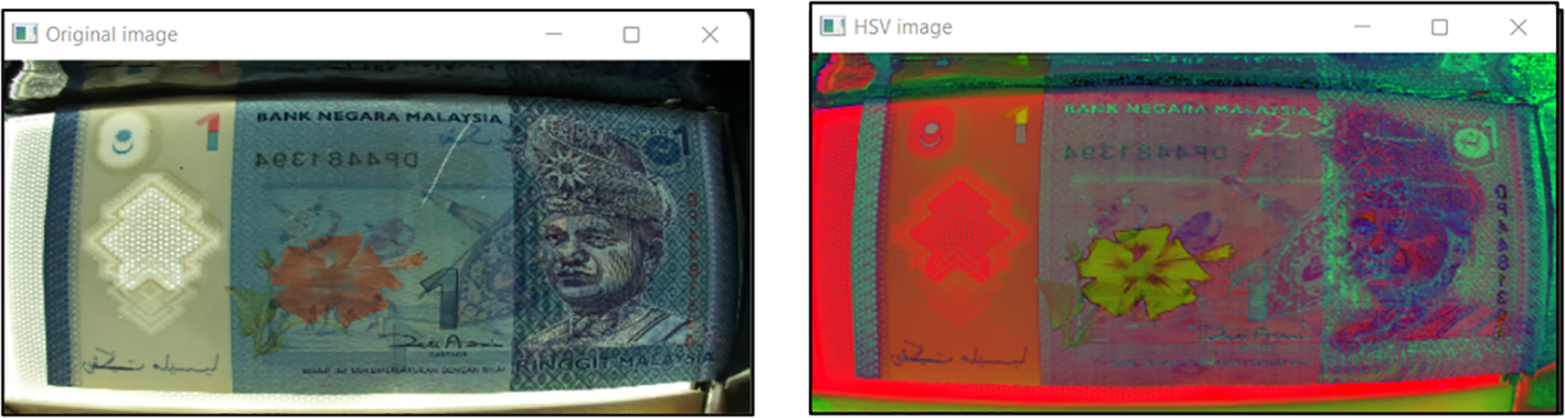
Original image (left) and HSV image (right).

Detect the clear window of RM1 or RM5 Using Mask detection algorithm. ^11^ HSV colour space is more often used in computer vision owing to its superior performance compared to RGB colour space in varying illumination levels. Thresholding and masking is done in HSV colour space. Figure 7 illustrates Hue, Saturation, Value (HSV) colour model and Figure 8, shows both the original image of RM1 and the converted image in HSV.

**Figure 9.**
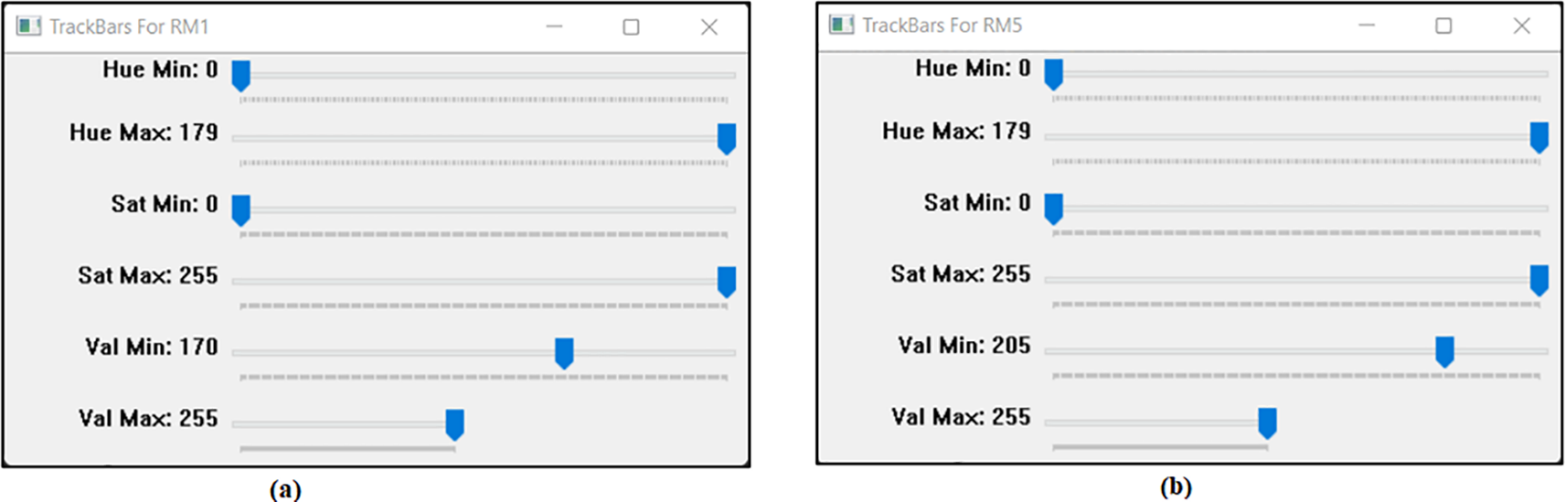
Track bars detect features in images.

The threshold values are fixed according to the banknote reader box internal environment and the front-backlight intensity. There are two set of threshold values set, one set for RM1 and another set for RM5. For RM1 the HSV value for the raspberry pi processor is fixed at Hue Min=0, Hue Max=179, Sat Min= 0, Sat Max=255, Val Min=170,Val Max=255 . For RM5 the HSV value for the raspberry pi processor is fixed at Hue Min=0, Hue Max=179, Sat Min=0, Sat Max=255, Val Min=205,Val Max=255. Figure 9(a) will show Track bars detect features in RM1 images. Figure 9(b) will show Track bars detect features in RM5 images.

**Figure 10.**
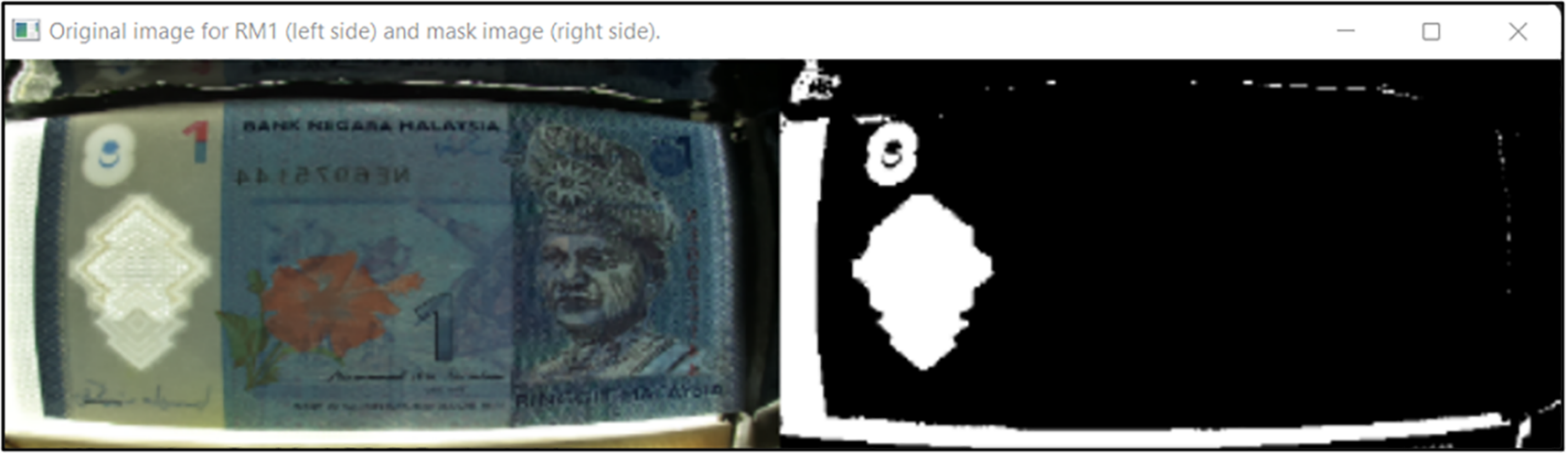
Original image for RM1 (left side) and mask image (right side).

**Figure 11.**
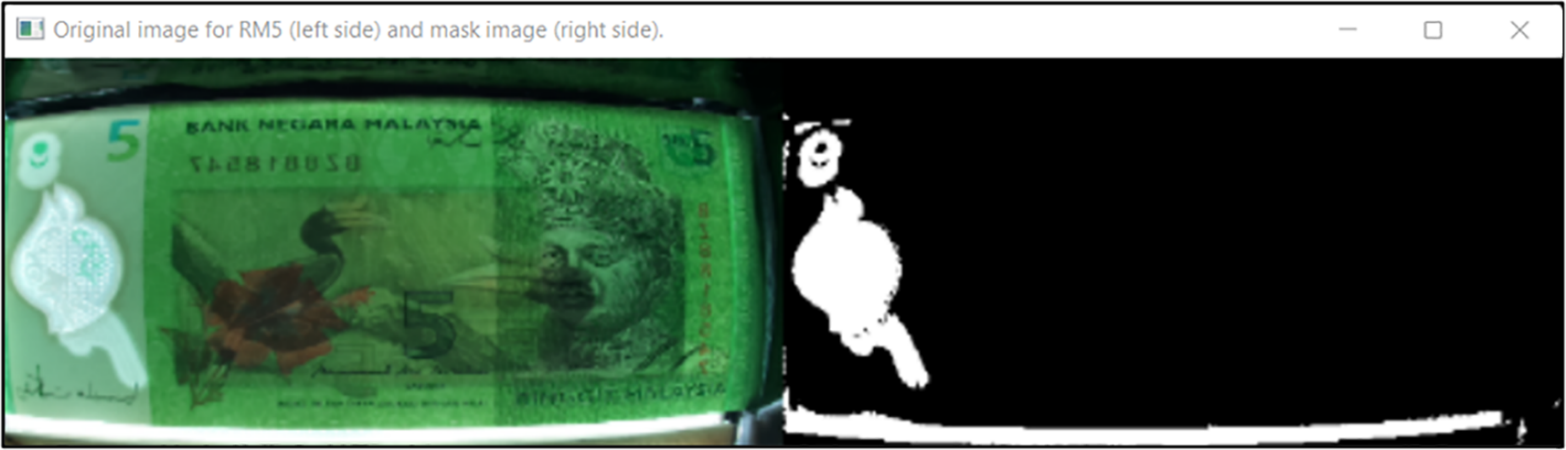
Original image for RM5 (left side) and it’s mask image (right side).

Specify the upper and lower bounds of the pixel’s values in the captured images. Figure 9 shown the track bars in python programming used to detect the features in images “Ba” and “Bb”. The set track bars HSV values will be used for the overall banknote detection later on. Figure 10 shown the original image for RM1 and its corresponding mask image. Figure 11 shown original image for RM5 and its corresponding mask image respectively.

If neither “Songket” nor “Hornbill” clear window is detected, then “the banknote is neither 1 Ringgit nor 5 Ringgit”.

Step 4: Three Regions of interest detection

Detect the three regions of interest, namely: Region1 (for transparent see thru window), Region 2 (for crescent and star) and Region 3 (for see-thru register). If clear window (white area in the red box Mask image as shown in Figure 10 for RM1 and Figure 11 for RM5) is detected, in the same area of original image (image “Bb”):

**(i)** Detect Regions of interest in RM1/RM5 banknote:

Search for the biggest and brightest/whitest bounded object, mark it as Region 1 (preparation for “Songket”/ “Hornbill” searching in Step 5). Then in the same clear window area of image “Bb”, search for the second biggest and brightest/whitest bounded object, mark it as Region 2 (preparation for “Crescent and Star” object pair searching in Step 5).

If Region 2 fall on the left side of the Y-axis symmetrical centreline of Region 1, then locate Region 3 at the right side with respect to the Y-axis symmetrical centreline of Region 1, by an area of ½ Region 1’s horizontal length in square’s dimension. Else if Region 2 fall on the right side of the Y-axis symmetrical centreline of Region 1, then locate Region 3 at the left side with respect to the Y-axis symmetrical centreline of Region 1, by an area of ½ Region 1’s horizontal length in square’s dimension.

**Figure 12.**
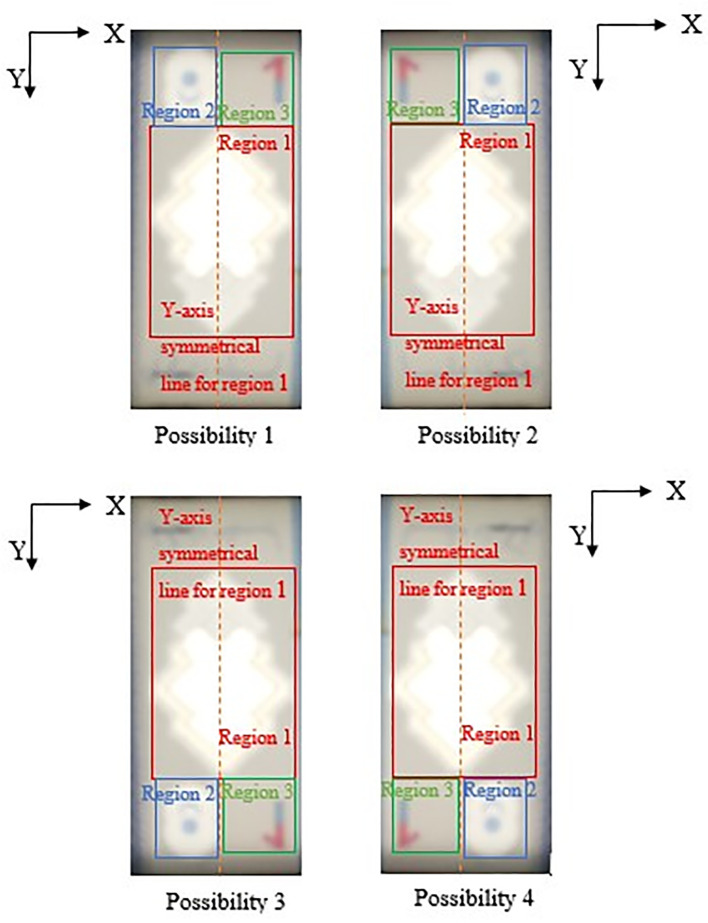
Four possibilities of RM1 correct detected 3 Region of interest.

**Figure 13.**
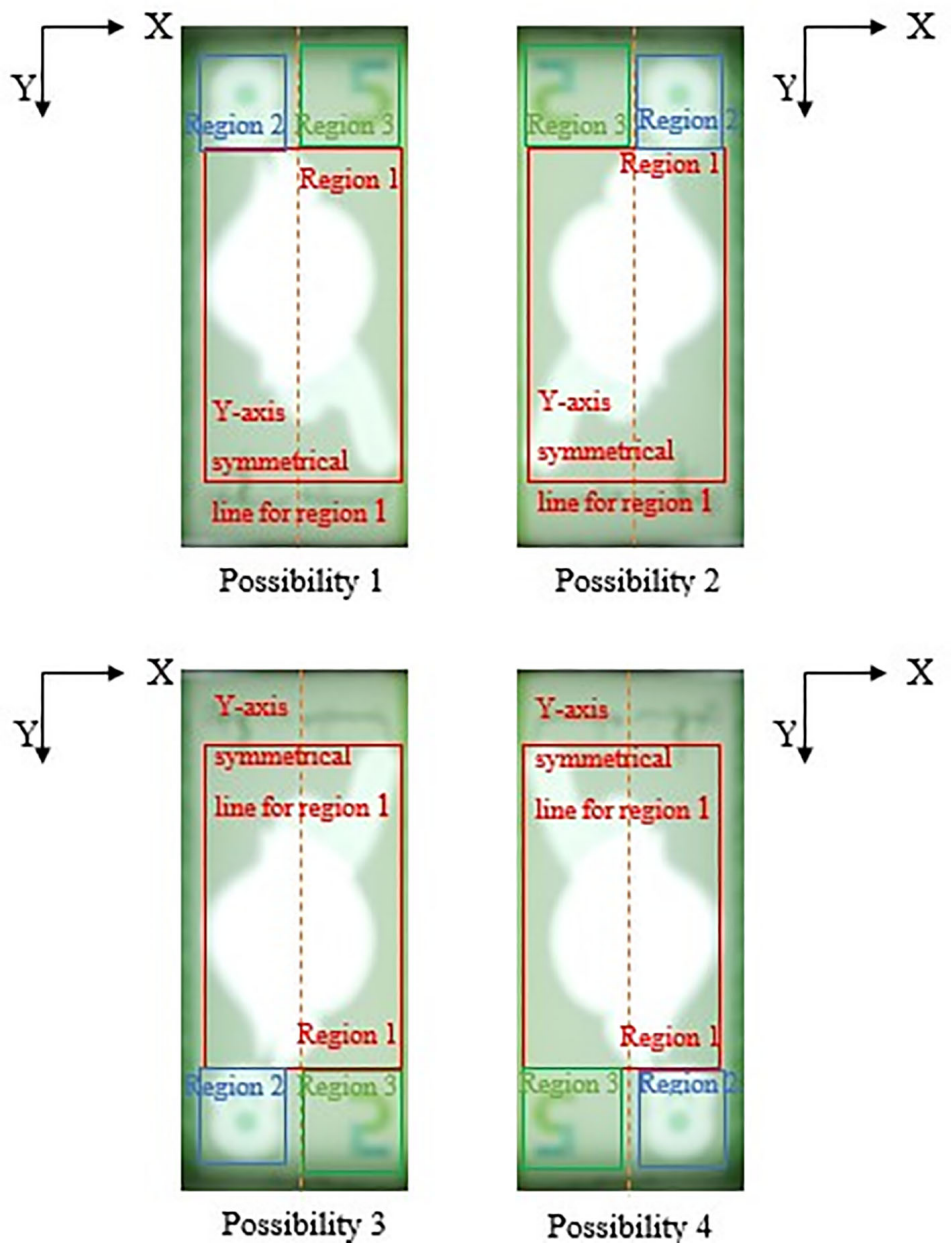
Four possibilities of RM5 correct detected 3 Region of interest.

Due to the reason that user might slot in banknotes into the banknote reader in different direction, the four possibilities of correct detected 3 Regions of interests for the slot in banknotes are shown in Figure 12 and Figure 13 below (For RM1 and RM5 respectively).

The reason that Region 2 is not similar size with Region 3 is because in RM1/RM5’s banknote design, portion numeric text (“1”/“5”) of the see-thru register fall in Region 3 might be clipped, rendering the watermark undetected if similar Region 2’s dimension is used for locating Region 3. Hence Region 3’s area should be assigned slightly bigger than Region 2.

**(ii)** Synchronize Regions of interest for better watermark detection in Step 5:

**–** Convert Possibility 2 case into Possibility 1 case

 IF Region 1’s Y-coordinates > Region 2’s Y-coordinates

  AND Region 2’s X-coordinates > Region 3’s X-coordinates,

  THEN “flipped image “Bb” horizontally, identify Region 1, 2 and 3 again using step (i) or step (ii) above”.

**–** Convert Possibility 3 case into Possibility 1 case

 IF Region 1’s Y-coordinates < Region 2’s Y-coordinates

  AND Region 2’s X-coordinates < Region 3’s X-coordinates,

  THEN “flipped the image “Bb” vertically, identify Region 1, 2 and 3 again using step (i) or step (ii) above”.

**–** Convert Possibility 4 case into Possibility 1 case

 IF Region 1’s Y-coordinates < Region 2’s Y-coordinates

  AND Region 2’s X-coordinates > Region 3’s X-coordinates,

  THEN “Performs image 180° rotation on the image “Bb”, identify Region 1, 2 and 3 again using step (i) or step (ii) above”.

Step 5: Watermarks detection

Detect the watermarks characteristics within each of the detected regions of interest.

(i) Region 1 detection:

Noise object exclusion: Check if the total pixels within the bounded area of the region,

$${\mathrm{TP}}_{R1}\geq P_{R1}\times \mathrm{TR}$$ (2)

where *P*_*R*1_ = Percentage of songket/hornbill area in a Malaysian Banknote.

*TR* = Total Pixels in the Resized Image converted in Step 2.

IF condition in equation (2) is NOT FULFILLED, Region 1 object is a noise object,

  THEN Output: “Region 1 watermark is not detected.”

ELSE IF condition in equation (2) is FULFILLED,

  THEN Region 1 object is a possible watermark, proceed to the below Bounding Box measurement.

**Figure 14.**
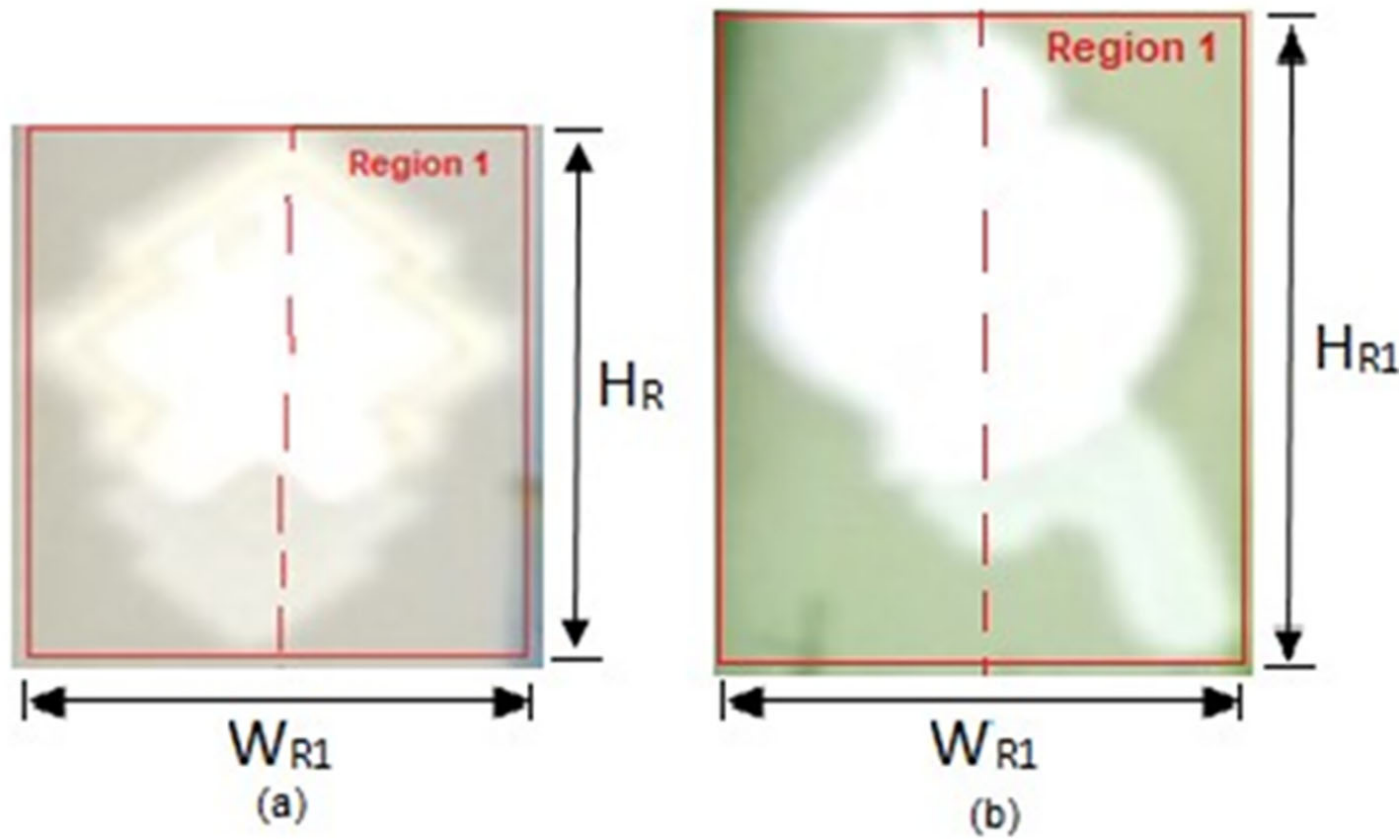
Bounding box of Region 1 (a) sample of RM1 (b) sample of RM5.

Bounding box measurement:

Assign *H*_*R*1_ as the height of the Region 1 bounding box *W*_*R*1_ as the width of the Region 1 bounding box (as shown in Figure 14 below).

Measure Region 1 bounding box’s height to width

$$\text{ratio}=H_{R1}/W_{R1}$$ (3)

IF *Th*_*R*1, *RM*1( *min*)_ < *H*_*R*1_/ *W*_*R*1_ < *Th*_*R*1, *RM*1( *max*)_, THEN Output: “Region 1’s watermark for RM1 is detected.”

ELSE IF *Th*_*R*1, *RM*5( *min*)_ < *H*_*R*1_/ *W*_*R*1_ < *Th*_*R*1, *RM*5( *max*)_,

  THEN Output: “Region 1’s watermark for RM5 is detected.”

ELSE Output: “Region 1’s watermark is not detected.”

where

**–** *Th*_*R*1, *RM*1( *min*)_ is the minimum threshold of RM1’s “Songket” height to width ratio

**–** *Th*_*R*1, *RM*1( *max*)_ is the maximum threshold of RM1’s “Songket” height to width ratio.

**–** *Th*_*R*1, *RM*5( *min*)_ is the minimum threshold of RM5’s “Hornbill” height to width ratio.

**–** *Th*_*R*1, *RM*5( *max*)_ is the maximum threshold of RM5’s “Hornbill” height to width ratio.

**Figure 15.**
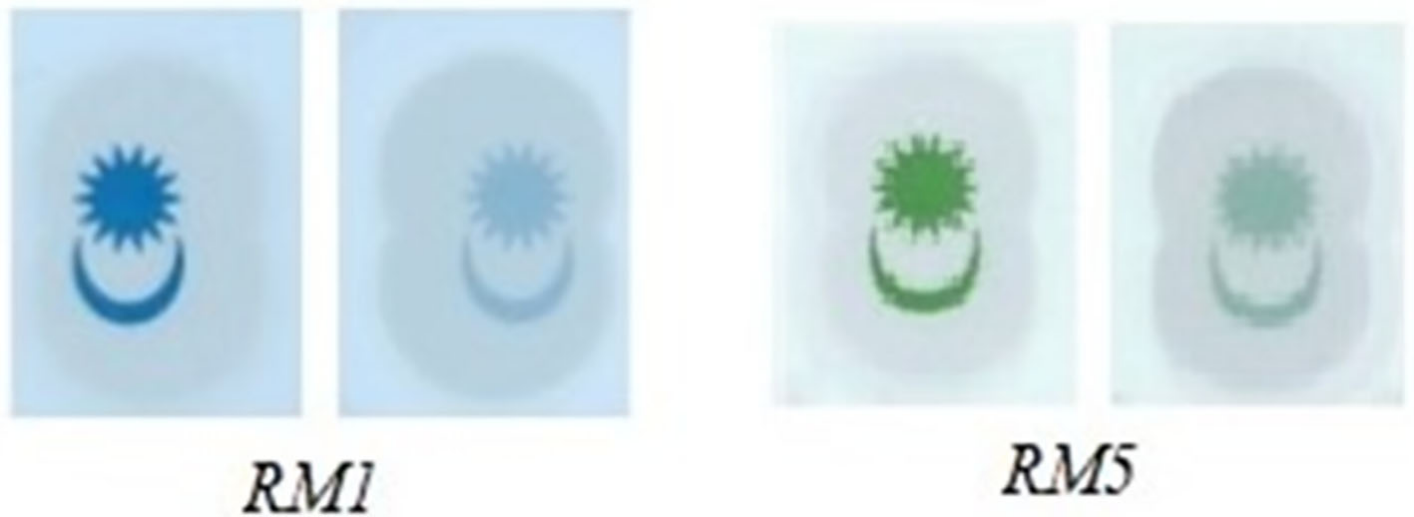
Sample of RM1 and RM5’s Crescent and Star images captured with backlight Off (left side) and with backlight On (right side).

**(ii)** Region 2 detection:

Compare the colour intensity of the Crescent and Star’s pixels in image “Ba” and image “Bb” (sample of RM1 and RM5 Crescent and Star are shown in Figure 15).

IF “Region 1’s watermark for RM1 is detected” AND *Th*_*R*2, *RM*1( *min*)_ <|Blue component for sampled pixel of Crescent and Star in image “Ba” - The same coordinate sampled pixel of Crescent and Star in image “Bb”|< *Th*_*R*2, *RM*1( *max*)_,

  THEN Output: “Region 2 watermark for RM1 is detected.”

ELSE IF “Region 1’s watermark for RM5 is detected” AND *Th*_*R*2, *RM*5( *min*)_ <|Green component for sampled pixel of Crescent and Star in image “Ba” - The same coordinate sampled pixel of Crescent and Star in image “Bb”|< *Th*_*R*2, *RM*5( *max*)_,

  THEN Output: “Region 2 watermark for RM5 is detected.”

ELSE Output: “Region 2’s watermark is not detected.” where

**–** *Th*_*R*2, *RM*1( *min*)_ is the minimum threshold of the acceptable colour intensity change of RM1’s “Crescent and Star” between the banknote image captured with backlight On (“Bb”) and backlight Off (“Ba”).

**–** *Th*_*R*2, *RM*1( *max*)_ is the maximum threshold of the acceptable colour intensity change of RM1’s “Crescent and Star” between the banknote image captured with backlight On (“Bb”) and backlight Off (“Ba”).

**–** *Th*_*R*2, *RM*5( *min*)_ is the minimum threshold of the acceptable colour intensity change of RM5’s “Crescent and Star” between the banknote image captured with backlight On (“Bb”) and backlight Off (“Ba”).

**–** *Th*_*R*2, *RM*5( *max*)_ is the maximum threshold of the acceptable colour intensity change of RM5’s “Crescent and Star” between the banknote image captured with backlight On (“Bb”) and backlight Off (“Ba”).

**(iii)** Region 3 detection:

Convert Region 3 in image “Ba” to Black and White image, name the new image as image “WBa”.

Convert Region 3 in image “Bb” to Black and White image, name the new image as image “WBb”.

Detect the numerical “1” or “5” in “WBa” and “WBb” using PyTesseract, ^13^ ^,^ ^14^ an OCR (optical character recognition) tool for python, which is the wrapper for Tesseract, ^15^ a free OCR engine sponsored by Google since 2006.

**Figure 16.**
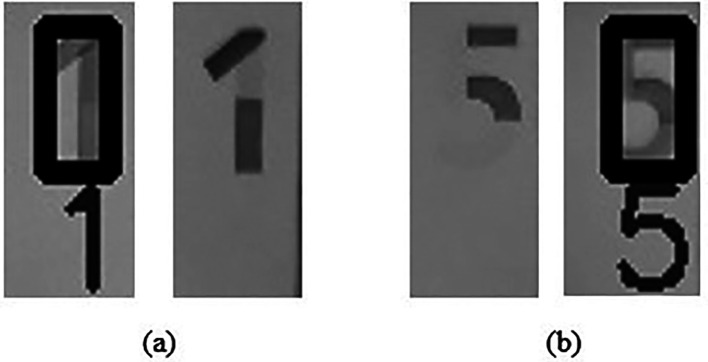
Sample of successful Region 3 detection (a) WBa and WBb for RM1 (b) WBa and WBb for RM5.

IF numerical “1” detected in image “WBb” AND not detected in image “WBa” (sample as shown in Figure 16a),

  THEN Output: “Region 3 watermark for RM1 is detected.”

ELSE IF numerical “5” detected in image “WBb” AND not detected in image “WBa” (sample as shown in Figure 16b),

  THEN Output: “Region 3 watermark for RM5 is detected.”

ELSE Output: “Region 3’s watermark is not detected.”

Step 6: Decision making

Apply fuzzy logic, T norms are used with AND connectors to make decision. The rules are set with at least 2 watermarks detected, only the banknote value is conforming and considered real. The fuzzy rules are set as below.

**(i).** FOR 1 RINGGIT.

**–** IF “Songket” clear window AND its corresponding Region 1, Region 2 AND Region 3 watermarks are detected, THEN the banknote is a REAL 1 RINGGIT.

**–** IF “Songket” clear window AND its corresponding Region 1 AND Region 2 watermarks are detected, THEN the banknote is a REAL 1 RINGGIT.

**–** IF “Songket” clear window AND its corresponding Region 1 AND Region 3 watermarks are detected, THEN the banknote is a REAL 1 RINGGIT.

**–** IF “Songket” clear window AND its corresponding Region 2 AND Region 3 watermarks are detected, THEN the banknote is a REAL 1 RINGGIT.

**(ii).** FOR 5 RINGGITS.

**–** IF “Hornbill” clear window AND its corresponding Region 1, Region 2 AND Region 3 watermarks are detected, THEN the banknote is a REAL 5 RINGGITS.

**–** IF “Hornbill” clear window AND its corresponding Region 1 AND Region 2 watermarks are detected, THEN the banknote is a REAL 5 RINGGIT.

**–** IF “Hornbill” clear window AND its corresponding Region 1 AND Region 3 watermarks are detected, THEN the banknote is a REAL 5 RINGGIT.

**–** IF “Hornbill” clear window AND its corresponding Region 2 AND Region 3 watermarks are detected, THEN the banknote is a REAL 5 RINGGIT.

**(iii).** FOR NOT A REAL BANKNOTE

**–** IF clear window is NOT detected, THEN the banknote is NOT a REAL BANKNOTE.

**–** IF Clear window is detected AND Region 1, 2 AND 3 watermarks are NOT detected, THEN the banknote is NOT a REAL BANKNOTE.

**–** IF Clear window is detected AND ONLY Region 1 watermark is detected, THEN the banknote is NOT a REAL BANKNOTE.

**–** IF Clear window is detected AND ONLY Region 2 watermark is detected, THEN the banknote is NOT a REAL BANKNOTE.

**–** IF Clear window is detected AND ONLY Region 3 watermark is detected, THEN the banknote is NOT a REAL BANKNOTE.

## 4. Experiment result

**Figure 17.**
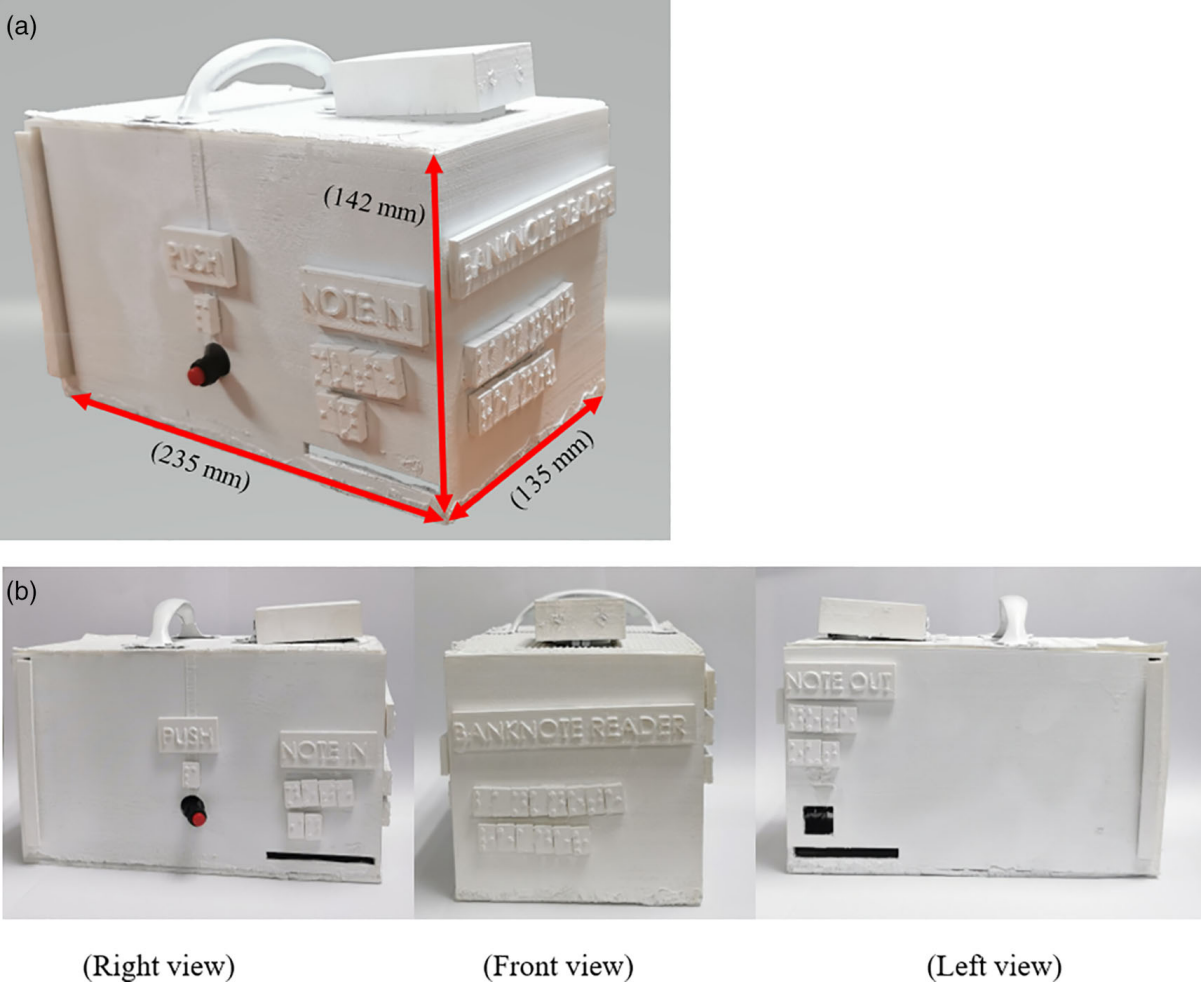
Banknote reader.

The prototype of RM1 and RM5 banknote reader is constructed, as shown in Figure 17. The dimension for the banknote reader prototype is 235 mm (Length) × 142 mm (Width) × 135 mm (Height). The filter size, or the standard deviation used for the Gaussian Blur function is 5×5 pixels. Such filter removed outlier 5×5 pixels that may be noise elements in the image.

Songket area in the real RM1 banknote is measured with dimension of 25 mm × 35 mm = 875 mm ^2^. The whole piece of RM1 banknote is with dimension 120 mm × 65 mm = 7,800 mm ^2^. Therefore, *P*_*R*1_ for RM1 is 11.22% or 0.1122. Hornbill area in the real RM5 banknote is measured with dimension of 25 mm × 40 mm = 1,000 mm ^2^. The whole piece of RM5 banknote is with dimension 135 mm × 65 mm = 8,775 mm ^2^. Therefore, *P*_*R*1_ for RM5 is 11.40% or 0.1140. Since the banknote reader is shared among RM1 and RM5 detection, hence the minimum *P*_*R*1_ among the two is selected, and rounded to 0.11.

TR is Total Pixels in the Resized Image converted in Step 2, *TR* = 250 (width) × 450 (height) = 112,500 pixels. Hence, in step 5 Noise Object Exclusion part, any object with bounding box region smaller than 0.11 × 112,500 = 12,375 pixels will not be considered as Region 1. The no. RGBchannels is 3.

**Figure 18.**
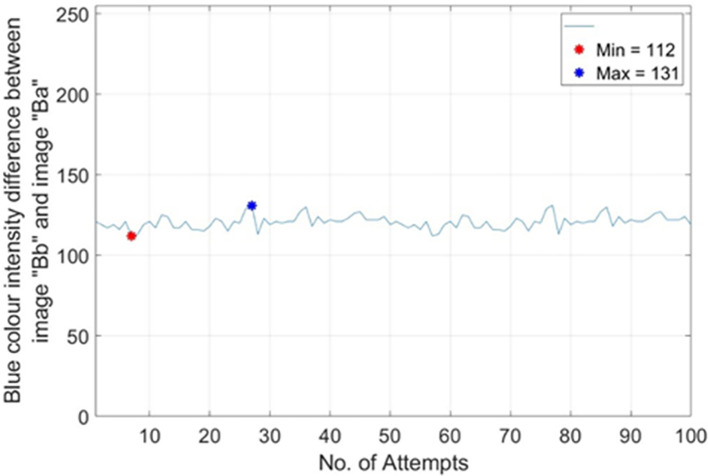
Plots on no. of attempts vs.|blue colour intensity.

The measured height of Songket’s pattern in RM1 banknote is 35 mm and the width of Songket’s pattern in RM1 banknote is 20 mm. Therefore, the height to width ratio of Songket’s pattern in RM1 banknote is 1.75. The measured height of Hornbill’s pattern in RM5 banknote is 43 mm and the width of Hornbill’s pattern in RM1 banknote is 23 mm. Therefore, the height to width ratio of Hornbill’s pattern in RM5 banknote is 1.87. To better classify RM1 and RM5 from one another, for RM1, *Th*_*R*1, *RM*1( *min*)_ is set to 1.69 and *Th*_*R*1, *RM*1( *max*)_ is set to 1.81; whereas for RM5, *Th*_*R*1, *RM*5 ( *min*)_ is set to 1.82 and *Th*_*R*1, *RM*5 ( *max*)_ is set to 1.93. Such setting is with the best tolerance gap to classify the two types of banknotes effectively. To get *Th*_*R*2, *RM*1_, 100 different real banknotes of RM1s’ images were captured for 100 pairs of image “Bb” (backlight On) and image “Ba” (backlight Off). The Blue colour intensity value on the Crescent and Star’s sampled pixels were recorded and the difference between image “Bb” and image “Ba” were calculated and tabulated in the plots of no. of attempts vs.|Blue colour intensity difference between image “Bb” and image “Ba”|as shown in Figure 18. From Figure 18, it is shown that most occurrence happened in between Blue colour intensity value of 112 to 131. Hence *Th*_*R*2, *RM*1( *min*)_ is set to 112 and *Th*_*R*2, *RM*1( *max*)_ is set to 131.

Difference between Image “Bb” and Image “Ba”|) for RM1

**Figure 19.**
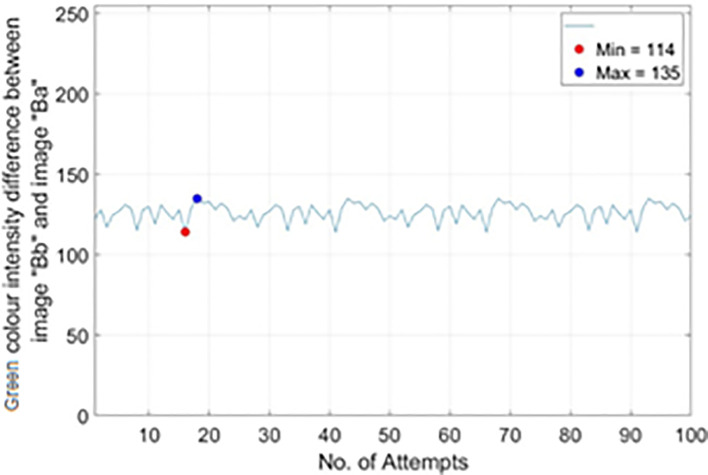
Plots on no. of attempts vs.|green colour intensity difference between image “Bb” and image “Ba”|) for RM5.

To get *Th*_*R*2, *RM*5_, 100 different real banknotes of RM5s’ images were captured for 100 pairs of image “Bb” (backlight On) and image “Ba” (backlight Off). The Green colour intensity value on the Crescent and Star’s sampled pixels were recorded and the difference between image “Bb” and image “Ba” were calculated and tabulated in the plots of no. of attempts vs. |Green colour intensity difference between image “Bb” and image “Ba”|as shown in Figure 19. From Figure 19, it is shown that most occurrence happened in between Green colour intensity value of 114 to 135. Hence *Th*_*R*2, *RM*5( *min*)_ is set to 114 and *Th*_*R*2, *RM*5( *max*)_ is set to 135.

**Figure 20.**
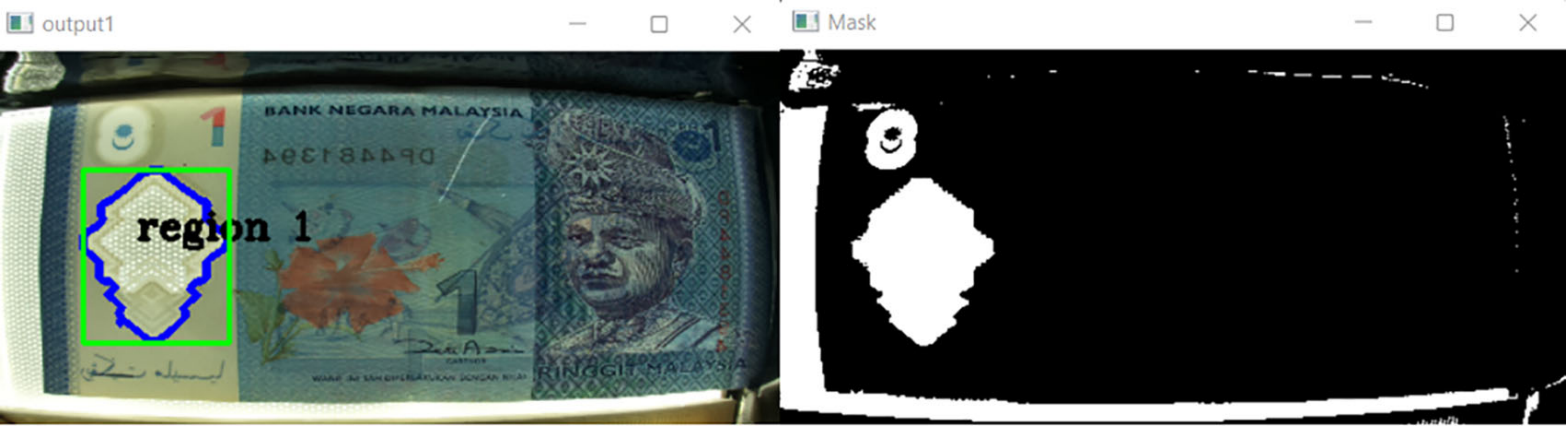
Real banknote RM1.

**Figure 21.**
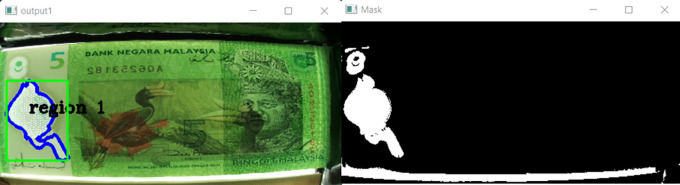
Real banknote RM5.

**Figure 22.**
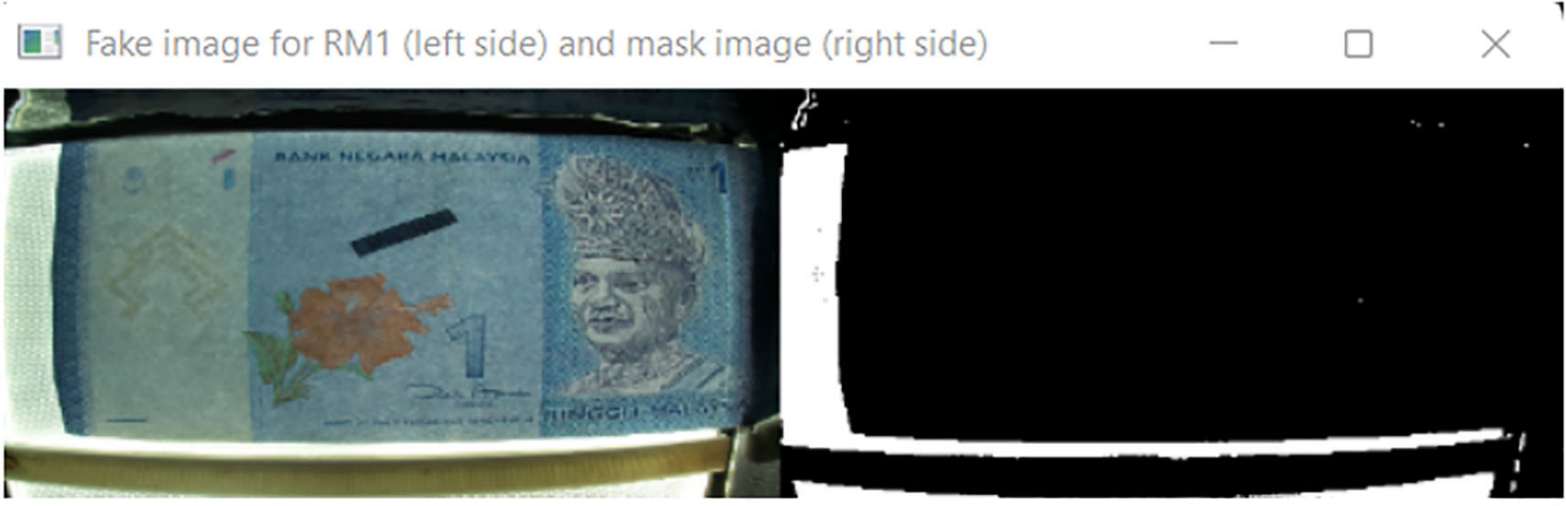
Fake banknote RM1.

**Figure 23.**
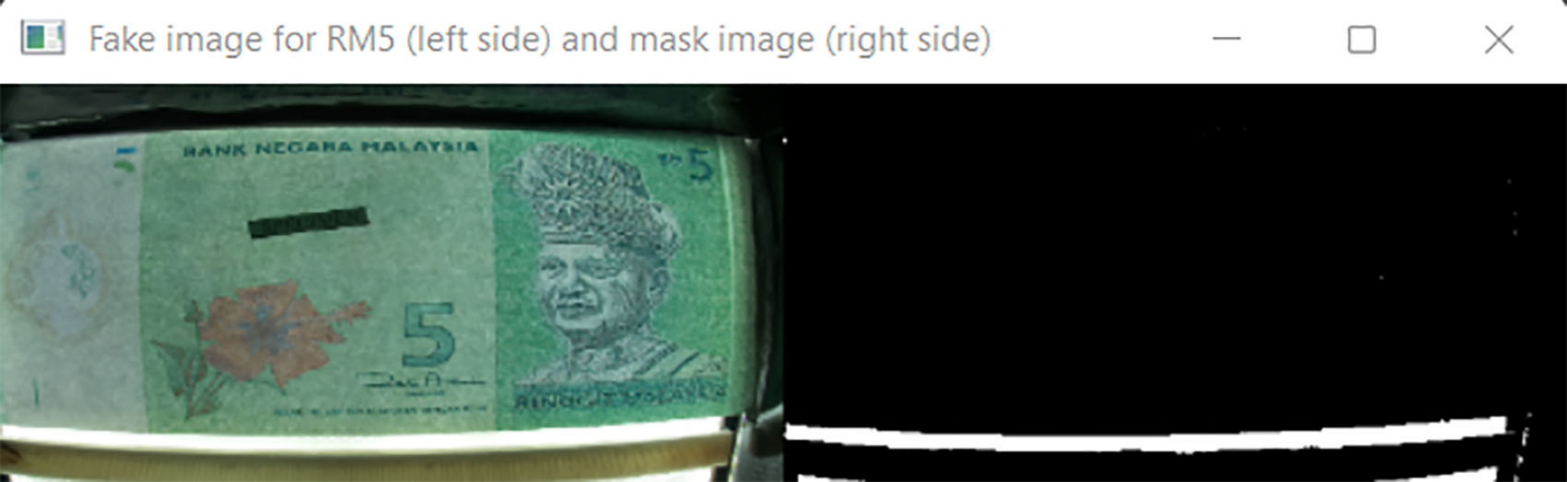
Fake banknote RM5.

In step 3 of the image processing algorithm, if the clear window of a songket (for 1 Ringgit) or a hornbill (for 5 Ringgit) can be detected, the banknote is genuine; otherwise, it is counterfeit. In mask, the HSV values of the colour that are filtered out. Figures 20– 23 illustrate the test run for some real and fake Malaysian banknotes. Experimental test was carried out with 100 pieces of real RM1, 100 pieces of real RM5 banknotes, 100 pieces of fake RM1 and 100 pieces of fake RM5 banknotes respectively revealed that the proposed banknote reader achieved around 99% accuracy for RM1 detection and around 78% accuracy for RM5 detection. The success rate of this system is up to 89% in recognizing the correct banknote value. From experimental test the threshold value of the acceptable colour intensity changes between the banknote image captured with and without backlight for RM1 ( *TH _B_*) from 41 to 57 and for RM5 ( *TH _G_*) from 60 to 78.

The total time for the banknote reader to complete 100 pieces of real RM1 banknotes detection is 1,148 seconds. Therefore, on average, the time required for one cycle of the banknote reader to capture in related banknote images, send to microcontroller to perform image processing and output the results on a speaker is 11.48 seconds.

Among the tested banknotes, for RM1, all the 100 pieces of the real banknotes and the 98 pieces of fake banknotes detected correctly. For RM5, 56 pieces of the real banknotes and all the 100 pieces of the fake banknotes detected correctly. To probe deep in to the failed banknote detection cases, confusion matrix is adopted. ^16^ The four possible outcomes for the banknote’s detection scenario are diagnosed as list in Table 1 and Table 2 for RM1 and RM5 respectively.

**Table 1.** Confusion matrix for RM1.

Position	Meaning
True positive (100)	The predicted RM1 banknote is real and it actually is real RM1 banknote.
True negative (98)	The predicted RM1 banknote is fake and it actually is fake RM1 banknote.
False positive (2)	The predicted RM1 banknotes is real and it actually is fake RM1 banknote.
False negative (0)	The predicted RM1 banknote is fake and it actually is real RM1 banknote.

**Table 2.** Confusion matrix for RM5.

Position	Meaning
True positive (56)	The predicted RM5 banknote is real and it actually is real RM5 banknote.
True negative (100)	The predicted RM5 banknote is fake and it actually is fake RM5 banknote.
False positive (0)	The predicted RM5 banknotes is real and it actually is fake RM5 banknote.
False negative (44)	The predicted RM5 banknote is fake and it actually is real RM5 banknote.

**Figure 24.**
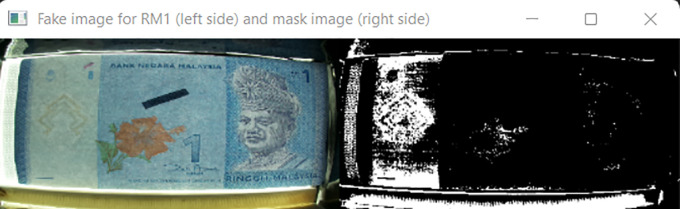
Sample of RM1 False Positive Case.

Noteworthy attentions are placed on False Positive and False Negative cases, because these two cases may cause the visually impaired person losing credits in their business. For RM 1 detection, 2 banknotes detection cases, related to False Positive class and none cases related to False Negative class. Further analysed on these 2 False Positive cases, it is found that the fake RM1 banknotes were not placed properly into the Malaysian banknote reader (center of the banknote slot) and the Malaysian banknotes reader had mistreated some other areas on the corresponding fake banknote as the three Region of interest area (as shown in Figure 24), and this further identified the fake RM1 as the real RM1. To overcome this problem, normalized sizes were assigned on RM1 and RM5 at the Step 2 Algorithm (resizing image portion) to better locking the three Region of interest area.

**Figure 25.**
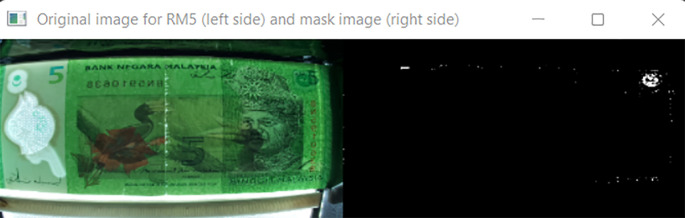
Sample of RM5 False Negative Case.

For RM5 detection, 44 cases related to False Negative class and none of the case relate to False Positive class. Further probed on these 44 False Negative Class cases, it is found out that majority of the captured “Bb” images were not fully covered, as shown in Figure 25. The slotted RM5 banknotes cannot fully picture by the imaging tool, causing some of the regions of interest on the inserted banknotes (especially Region 2 and Region 3) cannot be detected. This is due to the size of the RM5 is much bigger compare to RM1. To overcome this problem, imaging area for the inserted banknote should be increased to cover the full banknote’s image. However, with existing imaging tool, this might need to be tolerance with a longer focal length with bigger size of banknote reader. Another alternative is to search for a wide view imaging tool to replace the current imaging tool for optimizing the current Malaysian Banknotes Reader’s size.

Comparison of the proposed banknote reader detection accuracy and processing speed is done with three state-of-the-art methods: 1) VGG16 model using 2D Convolution Layer (32 neural) at TensorFlow's Keras API, ^17^ 2) MobileNet model using RMSprop Loss Function (learning_rate=0.0001) at TensorFlow's Keras API ^5^ and 3) Fuzzy Logic Based Perceptual Image Hashing Algorithm. ^18^

Experimental setup for method 1: Total of one hundred RM1 banknotes and one hundred RM5 banknotes are captured as the dataset for training the model. VGG16 model using 2D Convolution Layer (32 neural) at TensorFlow's Keras API being trained and tested with 100 real RM1, 100 real RM5, 100 fake RM1 and 100 fake RM5. The average time to load the model and build up the interpreter objects (Training time) was 60 seconds with batch size=32 and epochs=20 and the average inference time while modeling detecting banknote (Testing time) was 1 second. The test Accuracy was 60%.

Experimental setup for method 2: It is understood that the model MobileNet with Loss Function RMSProp was Selected as best accuracy of about 96.80% in paper. ^18^ Convolutional Neural Networks using MobileNet model with Loss Function RMSProp (0.0001) optimization technique being trained with one hundred RM1 banknotes and one hundred RM5 banknotes and tested with 100 real RM1, 100 real RM5, 100 fake RM1 and 100 fake RM5. The average time to load the model and build up the interpreter objects (Training time) was 81 seconds with batch size=32 and epochs=20 and the average inference time while modeling detecting banknote (Testing time) was 1 second. The test Accuracy was 50%.

Experimental setup for method 3: following paper. ^18^ algorithm. Fuzzy Logic Based Perceptual Image Hashing Algorithm first sorting Database using Perceptual Hashing with one hundred RM1 banknotes and one hundred RM5 banknotes and tested with 100 real RM1, 100 real RM5, 100 fake RM1 and 100 fake RM5. The average time to load the model and build up the interpreter objects (test 100 banknotes) was 130 seconds and the average inference time while detecting banknote (Per banknote) was 1.30 seconds. The test Accuracy was 42%.

The accuracy and required processing time for the experimented methods were summarized in Table 3. By comparing the above works on different Ringgit recognizers, it is observed that Fuzzy logic based light intensity variation watermark detection algorithm required longest processing time (both training and detection times for details watermark features extraction), however it has the best accuracy in detecting fake banknotes (minimum false positive and false negative cases) among the compared state-of-the-art methods. The VGG16 model, MobileNet model and Fuzzy Logic Based Perceptual Image Hashing Algorithm managed to be trained and detected the banknotes currency faster but with limitation of unable to accurately detecting fake banknotes (high false positive and false negative cases recorded) due to no watermarks detection consideration.

**Table 3.** Experimental results for accuracy and processing time with three state-of-the-art methods

Accuracy						Processing time	
		True positive	True negative	False positive	False negative	System training time (per 100 banknotes)	Banknote detection time (per banknote)
**Fuzzy logic based light intensity variation watermark detection algorithm**	RM1	100	98	2	0	1,148 Second	11.48 Second
	RM5	56	100	0	44	1,148 Second	11.48 Second
**VGG16 model using 2D Convolution Layer (32 neural) at TensorFlow's Keras API**	RM1	100	0	100	0	60 Second	1 Second
	RM5	100	40	60	0		
**MobileNet model using RMSprop Loss Function (learning_rate=0.0001) at TensorFlow's Keras API**	RM1	100	0	100	0	81 Second	1 Second
	RM5	100	0	100	0		
**Fuzzy Logic Based Perceptual Image Hashing Algorithm**	RM1	90	21	79	10	130 Second	1.30 Second
	RM5	56	0	100	44	130 Second	1.30 Second

## 5. Conclusions

A Malaysian banknote reader employing image processing techniques was developed for visually impaired person to read and identify counterfeit on one Ringgit and five Ringgit Malaysian banknotes. The proposed portable banknote reader employed a visual type sensor to capture the inserted banknote image, sent to a Raspberry Pi controller for extracting the banknote’s watermarks and identify the banknote’s value. The detection result will be broadcasted on a mini speaker mounted on the banknote reader to help the visually impaired comprehend if it is a real one Ringgit, real five Ringgit, or none of them. The experimental results had proven that the proposed banknote reader is capable of completing several rounds of successful tries. In future, tilting/rotating mechanism and Ultraviolet light shooting mechanism can be embedded on the banknote reader to allow the visually impaired persons to cover the full series of Malaysian banknotes reading capabilities. The Malaysian banknote reader can also be expanded to support additional foreign currencies reading in the future. Aside from that, the size of the banknote reader can be improved, as well as the classifier intervention in the banknote interpretation. These issues will be resolved in the future.

## Data availability

All data underlying the results are available as part of the article and no additional source data are required.
