# Supplementary material for: One- and Five-Ringgit Malaysia banknotes reader with counterfeit detection for visually impaired person using backlight mechanism and image processing techniques
Source: F1000Res. 2022 Apr 13;10:1098. Originally published 2021 Oct 29. [Version 2] doi: 10.12688/f1000research.58446.2 (PMC11009568; doi:10.12688/f1000research.58446.2)
Supplement: Peer review history

# Reviewer report: Reviewer response for version 2

**DOI:** 10.5256/f1000research.125690.r256677

**Published:** 2024-04-11

**Authors:** Rana Mohtasham Aftab

**Affiliations:** 1 Lahore Leads University, Lahore, Punjab, Pakistan; 2 Computer science, University of engineering and technology lahore, Lahore, Pakistan, Pakistan

The research article presents an innovative solution addressing the challenges faced by visually impaired individuals in handling banknotes, particularly in recognizing their value and detecting counterfeit. The proposed Malaysian banknote reader integrates image processing techniques with a portable design to offer real-time assistance to users.

 One notable aspect of this study is the emphasis on addressing both recognition and counterfeit detection functionalities. While existing solutions focus solely on recognizing banknote values, this research fills a crucial gap by incorporating counterfeit detection features. By leveraging image processing algorithms and watermarks extraction, the proposed banknote reader offers a comprehensive solution to assist visually impaired individuals in differentiating between genuine and counterfeit Malaysian banknotes.

 The experimental results showcased the efficacy of the developed banknote reader, demonstrating successful outcomes across multiple trials. The utilization of a Raspberry Pi controller for image processing and value identification, coupled with a mini speaker for broadcasting results, enhances accessibility for visually impaired users. Moreover, the incorporation of a visual type sensor ensures ease of use and portability, catering to the needs of users in various settings.

 The paper also highlights avenues for future research and improvement. Suggestions such as integrating tilting/rotating mechanisms and ultraviolet light shooting for broader currency coverage showcase a forward-thinking approach towards enhancing the capabilities of the banknote reader. Additionally, the potential expansion to support foreign currencies reading underscores the scalability of the proposed solution.

 However, the review could benefit from further elaboration on certain aspects. For instance, additional insights into the methodology employed for image processing and counterfeit detection would enhance the understanding of the proposed approach. Furthermore, a discussion on the potential limitations or challenges encountered during the experimental phase could provide valuable context for the reader.

 Overall, the research article presents a significant contribution to assistive technology for visually impaired individuals. By combining recognition and counterfeit detection features in a portable banknote reader, the study offers a practical solution to address the unique needs of users in handling Malaysian banknotes. The proposed enhancements and future directions outlined in the paper lay a solid foundation for further advancements in this field, with the potential to benefit a wider audience beyond the scope of the current study.

Is the work clearly and accurately presented and does it cite the current literature?

Yes

If applicable, is the statistical analysis and its interpretation appropriate?

Yes

Are all the source data underlying the results available to ensure full reproducibility?

Yes

Is the study design appropriate and is the work technically sound?

Yes

Are the conclusions drawn adequately supported by the results?

Yes

Are sufficient details of methods and analysis provided to allow replication by others?

Yes

Reviewer Expertise:

Machine learning

I confirm that I have read this submission and believe that I have an appropriate level of expertise to confirm that it is of an acceptable scientific standard.

# Reviewer report: Reviewer response for version 2

**DOI:** 10.5256/f1000research.125690.r134738

**Published:** 2022-04-21

**Authors:** Haidi Ibrahim

**Affiliations:** 1 School of Electrical and Electronic Engineering, Universiti Sains Malaysia, Nibong Tebal, Malaysia

I am satisfied with the improvements and justifications given by the authors.

Is the work clearly and accurately presented and does it cite the current literature?

Partly

If applicable, is the statistical analysis and its interpretation appropriate?

Yes

Are all the source data underlying the results available to ensure full reproducibility?

No

Is the study design appropriate and is the work technically sound?

Partly

Are the conclusions drawn adequately supported by the results?

Yes

Are sufficient details of methods and analysis provided to allow replication by others?

Partly

Reviewer Expertise:

NA

I confirm that I have read this submission and believe that I have an appropriate level of expertise to confirm that it is of an acceptable scientific standard.

# Reviewer report: Reviewer response for version 1

**DOI:** 10.5256/f1000research.62043.r122605

**Published:** 2022-02-21

**Authors:** Haidi Ibrahim

**Affiliations:** 1 School of Electrical and Electronic Engineering, Universiti Sains Malaysia, Nibong Tebal, Malaysia

There are some suggestions for the improvements of the manuscript (page numbers refer to the pdf version of the article):

1. To highlight the contribution of the work, it would be better to add terms “counterfeit detection” and “visually impaired person” to the title. For example, “One- and five-Ringgit Malaysia banknotes reader with counterfeit detection for visually impaired person using backlight mechanism and image processing techniques”.
2. Abbreviations should be defined properly.  For examples, what are SPB, IPPLEX and UTM on page 3?
3. On page 3, 2 ^nd^ last paragraph, the authors did mention that the available Malaysian banknote readers are huge in size. However, the system developed, as shown in Figure 15, also seems bulky. (It is also better if the labels and dimensions of Figure 15 are provided). Besides, if the authors want to show that the proposed system has advantage in terms of size, it would be nicer if there is a table to compare the size of the proposed system with the available systems.
4. In Section 1, it would be better if the authors could provide an introduction to RM1 and RM5 banknotes.  Better to provide figure(s) with labels (e.g., songket, hornbill) for this purpose.
5. Better to treat divisions in Section 2 as subsections, and given number, such as Section 2.1, Section 2.2, etc.
6. In Section 2, it would be better to discuss about the micro-controller first before the other components. Thus, it would be clearer, for example, why the authors are considering the use of Raspberry Pi cameras.
7. Section 3 is mostly in point form. A better presentation is needed. The authors could describe the methods in paragraphs, and explain with the help of figures, flowchart, or pseudocodes.
8. The method in Section 3 is not clear. For example, on page 6, in Figure 3, it is shown image “Bc”, but when image “Bc” is used for banknotes detection it is not mentioned clearly.
9. Page 7, 2 ^nd^ line. How can converting the RGB to grayscale image help in improving image quality and reduce image noise?
10. In equation (1), why are the input images (Ba and Bb) located on the left side of the equation, and not on the right side? Usually, the left side is for the output.
11. In equation (1), better to mention the values of “width”, “height”, and “no. RGBchannels” used in this work.
12. For the Gaussian Blur function, what is the filter size, or the standard deviation used?
13. Figure 4 does not show the Gaussian Blur converted image, but the image after grayscale conversion.
14. If the image is already converted to grayscale image, why should we convert it to HSV space? Or is the conversion from the RGB image? If this is from the RGB image, then why do we need to convert the image into grayscale? Besides, why we do not use “Bc” for this purpose?
15. If Figure 5 is taken somewhere, a proper permission should be asked to re-publish this figure. Citation should be given in the figure’s caption.
16. Figure 7 shows how the thresholding process is done by using track bars. The question is, are these threshold values fixed for all input images, or need to be changed, depending to the input image? If it is not fixed, then the method is not automated, and the user needs to set it every time a banknote is input to the system. Besides, is this process suitable for a visually impaired person?
17. Page 9, descriptions for part (i) and part (ii) are similar to each other.
18. Figure 15 should also label where the slot to input the banknote to the system, and where the banknote will exit from the system. 
19. The system has a speaker. What is the sound/notification given to the user? Some description on how to set up this sound/notification should be given.
20. In Section 1, more review on the related works should be done. For example:

- http://eprints.utar.edu.my/4289/1/17ACB01383_FYP.pdf
- https://www.hindawi.com/journals/js/2022/4505089/
- https://iopscience.iop.org/article/10.1088/1742-6596/2089/1/012008/meta

Is the work clearly and accurately presented and does it cite the current literature?

Partly

If applicable, is the statistical analysis and its interpretation appropriate?

Yes

Are all the source data underlying the results available to ensure full reproducibility?

No

Is the study design appropriate and is the work technically sound?

Partly

Are the conclusions drawn adequately supported by the results?

Yes

Are sufficient details of methods and analysis provided to allow replication by others?

Partly

Reviewer Expertise:

Digital image processing and analysis.  Digital signal processing and analysis.

I confirm that I have read this submission and believe that I have an appropriate level of expertise to state that I do not consider it to be of an acceptable scientific standard, for reasons outlined above.

## References

[1] : Malaysia currency recognizer mobile application for visual impairment. *Universiti Tunku Abdul Rahman, Malaysia* .2022; Reference source

[2] : Ethiopian Banknote Recognition Using Convolutional Neural Network and Its Prototype Development Using Embedded Platform. *Journal of Sensors* .2022;2022: 10.1155/2022/4505089 1-18 10.1155/2022/4505089

[3] : Indian Currency Denomination Recognition and Fake Currency Identification. *Journal of Physics: Conference Series* .2021;2089(1) : 10.1088/1742-6596/2089/1/012008 10.1088/1742-6596/2089/1/012008

# Response

**Published:** 2022-04-07

**Authors:** Wai Kit Wong

**Affiliations:** Multimedia University, Malaysia

**1. Reviewer :** To highlight the contribution of the work, it would be better to add terms “counterfeit detection” and “visually impaired person” to the title. For example, “One- and five-Ringgit Malaysia banknotes reader with counterfeit detection for visually impaired person using backlight mechanism and image processing techniques”.

**Author:** The title of the article is revised to “One- and Five-Ringgit Malaysia banknotes reader with counterfeit detection for visually impaired person using backlight mechanism and image processing techniques”.

**2. Reviewer :** Abbreviations should be defined properly.  For examples, what are SPB, IPPLEX and UTM on page 3?

**Author :** The abbreviations are well defined: SPB in Section 1 INTRODUCTION Paragraph 2 is replaced with full form “Seri Paduka Baginda”. IPPLEX is a company develops application services enabled by fixed and wireless IP networks and the convergence of media communications. UTM in Section 1 INTRODUCTION Paragraph 6 is added with full form “Universiti Teknologi Malaysia”.

**3. Reviewer 2:** On page 3, 2 ^nd^ last paragraph, the authors did mention that the available Malaysian banknote readers are huge in size. However, the system developed, as shown in Figure 15, also seems bulky. (It is also better if the labels and dimensions of Figure 15 are provided). Besides, if the authors want to show that the proposed system has advantage in terms of size, it would be nicer if there is a table to compare the size of the proposed system with the available systems.

**Author:** The proposed system is not having advantage in terms of size compared to some available Malaysian banknote reader in the market. Therefore, the issue of bulky size reader will not be discussed in the paper. Labels and dimensions of Figure 17 are provided 235 mm (Length) x142 mm (Width) x135 mm (Height), for readers to justify the banknote reader size themselves.

**4. Reviewer :** In Section 1, it would be better if the authors could provide an introduction to RM1 and RM5 banknotes.  Better to provide figure(s) with labels (e.g., songket, hornbill) for this purpose.

**Author:** An introductory paragraph of RM1 and RM5 banknotes is added in Section 1 INTRODUCTION Paragraph 3 (after "FEEL, LOOK, TILT, and CHECK" principle), together with Figure 1 with labels (songket, hornbill):

 The RM1 and RM5 Malaysian Banknotes are shown in Figure 1a and Figure 1b respectively. These banknotes are made from polymer substrate and with security features/watermarks with label 1-8. In sequence: 1) Intaglo, 2) Clear Window, 3) Shadow Image, 4) Crescent & Star Non-transparent window, 5) Perfect see thru Register, 6) Micro-Lettering, 7) Two color fluorescent element for Perfect see-thru, 8) UV BNM Text and Logo. Among the eight types of watermarks, there are three types of watermarks that related to the use of front-backlight mechanism ( 1)see-thru windows, 2)Crescent and Star non transparent window, 3)Perfect see though register). These three types of watermarks will be selected for the proposed prototype to run test.

 Added Figure 1 (a) RM1  (b) RM5 and their Corresponding watermarks.

**5. Reviewer :** Better to treat divisions in Section 2 as subsections, and given number, such as Section 2.1, Section 2.2, etc.

**Author:** Divisions in Section 2 are treated as subsections e.g: Section 2.1 Micro-controller, Section 2.2 Imaging Tools, Section 2.3 Backlight Platform, Section 2.4 Speaker and Section 2.5 Battery.

**6. Reviewer :** In Section 2, it would be better to discuss about the micro-controller first before the other components. Thus, it would be clearer, for example, why the authors are considering the use of Raspberry Pi cameras.

**Author:** Revised Section 2, micro-controller is discussed first (moved to Section 2.1) before the other components.

 7. **Reviewer :** Section 3 is mostly in point form. A better presentation is needed. The authors could describe the methods in paragraphs, and explain with the help of figures, flowchart, or pseudocodes.

**Author:** Revised as per in Reviewer 1’s Comment 3.

**8. Reviewer :** The method in Section 3 is not clear. For example, on page 6, in Figure 3, it is shown image “Bc”, but when image “Bc” is used for banknotes detection it is not mentioned clearly.

**Author:** Image “Bc” and its related definition is removed from the text to avoid confusion.

**9. Reviewer :** Page 7, 2 ^nd^ line. How can converting the RGB to grayscale image help in improving image quality and reduce image noise?

**Author:** The numeral printed on the banknote can be easily traced out by optical character recognition software in grayscale format. 

**10. Reviewer :** In equation (1), why are the input images (Ba and Bb) located on the left side of the equation, and not on the right side? Usually, the left side is for the output.

**Author:** The equation actually carried the meaning of the new Ba and Bb images will be resized to the desired width; height and number of RGBchannel after execution.

**11. Reviewer :** In equation (1), better to mention the values of “width”, “height”, and “no. RGBchannels” used in this work.

**Author:** The values of “width”, “height”, and “no. RGBchannels” used in this work are selected in Section 4 Paragraph 4 (TR = 250(width) x 450(height) = 112,500 pixels). The no. RGBchannels is 3. Added in Section 4 Paragraph 4.

 12. **Reviewer :** For the Gaussian Blur function, what is the filter size, or the standard deviation used?

****

**Author:** The filter size, or the standard deviation used for the Gaussian Blur function is 5x5 pixels. Such filter removed outlier 5x5 pixels that may be noise elements in the image. Added in Section 4 Paragraph 1. 

 13. **Reviewer :** Figure 4 does not show the Gaussian Blur converted image, but the image after grayscale conversion.

**Author:** Figure 6 added a Gaussian Blur converted image.

**14. Reviewer :** If the image is already converted to grayscale image, why should we convert it to HSV space? Or is the conversion from the RGB image? If this is from the RGB image, then why do we need to convert the image into grayscale? Besides, why we do not use “Bc” for this purpose?

**Author:** To extract region 1 the RGB image “Bb” needs to convert to a Gaussian Blur image and further convert to an HSV space image. However, to extract region 3 the RGB image “Ba” and “Bb” need to convert to grayscale images.

**15. Reviewer **: If Figure 5 is taken somewhere, a proper permission should be asked to re-publish this figure. Citation should be given in the figure’s caption.

**Author:** Figure 7 is taken from this paper: Vazquez Saraullo, Federico Alejandro & Larosa, Facundo & Ghignone, Ramiro & Lanzillotta, Lucas. (2019) “Diseño, implementación y ensayo de un lector de colores de bajo costo para personas ciegas y disminuidas visuals”, X Congreso de Microelectrónica Aplicada (μEA2019)At: San Martín, Buenos Aires, Argentina. Citation for Figure 7 is done and listed in the paper with citation [18].

**16. Reviewer :** Figure 7 shows how the thresholding process is done by using track bars. The question is, are these threshold values fixed for all input images, or need to be changed, depending to the input image? If it is not fixed, then the method is not automated, and the user needs to set it every time a banknote is input to the system. Besides, is this process suitable for a visually impaired person?

**Author:**  Yes, the threshold values are fixed according to the banknote reader box internal environment and the front-backlight intensity. There are two set of threshold values set, one set  for RM1 and another set for RM5. For RM1 the HSV value for the raspberry pi processor is fixed at Hue Min = 0 , Hue Max = 179, Sat Min= 0, Sat Max=255, Val Min=170,Val Max=255 . For RM5 the HSV value for the raspberry pi processor is fixed at Hue Min = 0 , Hue Max = 179, Sat Min=0, Sat Max=255, Val Min=205,Val Max=255 . Figure 9 (a) will show Track bars detect features in RM1 images. Figure 9 (b) will show Track bars detect features in RM5 images. This detail explanation is added in Section 3 Step 3 Second paragraph.

**17. Reviewer :** Page 9, descriptions for part (i) and part (ii) are similar to each other.

**Author: **In Section 3, Step 4 description for part (i) and part (ii) are similar to each other. Part (i) is detecting Region of Interest for RM1 whereas Part (ii) is detecting Region of Interest for RM5. Hence the two parts are combined to become one part.

**18. Reviewer :** Figure 15 should also label where the slot to input the banknote to the system, and where the banknote will exit from the system. 

**Author:** Figure 17 revised to label where the slot to input the banknote to the system, and where the banknote will exit from the system.

**19. Reviewer :** The system has a speaker. What is the sound/notification given to the user? Some description on how to set up this sound/notification should be given.

**Author:** The notification messages given to the users include: “Real one Ringgit”, “Real five Ringgit” and “Not a Malaysian banknote.”

 Description : Import pyttsx3 library in Python. It is a a text-to-speech conversion library in Python. The results in step 6 Decision making part will be sent to activate the text (eg. Real one Ringgit, Real five Ringgit or Not a Malaysian banknote.” The pyttsx3 command will transfer the text to speech and display at the speaker. Below is the sample of codings:

 engine = pyttsx3.init()

 engine.say("Real five ringgit")

 engine.runAndWait()

 engine.stop()

 Added in the Section 2.4 Speaker

**20. Reviewer :** In Section 1, more review on the related works should be done. For example:

- http://eprints.utar.edu.my/4289/1/17ACB01383_FYP.pdf

1. Lee KL: Malaysia currency recognizer mobile application for visual impairment. Universiti Tunku Abdul Rahman, Malaysia. 2022. Reference Source

- https://www.hindawi.com/journals/js/2022/4505089/

2. Aseffa D, Kalla H, Mishra S: Ethiopian Banknote Recognition Using Convolutional Neural Network and Its Prototype Development Using Embedded Platform. Journal of Sensors. 2022; 2022: 1-18 Publisher Full Text

- https://iopscience.iop.org/article/10.1088/1742-6596/2089/1/012008/meta

3. Padmaja B, Naga Shyam Bhargav P, Ganga Sagar H, Diwakar Nayak B, et al.: Indian Currency Denomination Recognition and Fake Currency Identification. Journal of Physics: Conference Series. 2021; 2089 (1). Publisher Full Text

         

**Author:** The review of the THREE (3) works above are considered and added in Section 1 Paragraph 8 (After NantMobile): Convolutional Neural Networks using MobileNet model was selected by [15] in detecting Ethiopian banknotes. Convolutional Neural Networks using Canny Edge detection and multiscale template matching methods were selected by [17] in detecting Indian banknotes. Both these two models are detecting banknotes denomination and counterfeit. However, their counterfeit detection only focus on banknotes’ surface security features, like micro-lettering and only can detect single - sided of banknotes. Unlike other hidden type of watermarks, micro-lettering is easy to be printed by current high-resolution printers.

# Reviewer report: Reviewer response for version 1

**DOI:** 10.5256/f1000research.62043.r120762

**Published:** 2022-01-25

**Authors:** Neeraj Dhanraj Bokde

**Affiliations:** 1 Aarhus University, Aarhus, Denmark

The manuscript presented an image processing technique for two Malaysian banknotes detection using a microcontroller-based mechanism. The research can have a good impact on society and is worth investigating, however, the research component of the manuscript is minimal. The authors may consider the following comments to revise the manuscript:

1. The research contribution and novelty in terms of image processing are not well discussed in the manuscript. It is advised to discuss the methodology proposed by the authors to solve the problem statement.
2. Besides, it is very crucial to compare the performance of the proposed methodology with the state-of-the-art methods and evaluate its performance in terms of different error metrics.
3. The author tried different IF-ELSE situations to detect the currency, however, the presentation of the same is very poor in the manuscript. It is advised to discuss these things in the form of block diagrams and Psuedo codes with proper formatting.
4. The quality of figures in terms of resolution and aesthetics are very poor. It is advised to revise all figures with improved qualities.
5. The manuscript in the present form is like a project report, and not suitable for a research article. It is recommended to revise the manuscript with an improved case study that will discuss the research contributions in more detail than the hardware-software interface systems.

Is the work clearly and accurately presented and does it cite the current literature?

Yes

If applicable, is the statistical analysis and its interpretation appropriate?

No

Are all the source data underlying the results available to ensure full reproducibility?

Partly

Is the study design appropriate and is the work technically sound?

No

Are the conclusions drawn adequately supported by the results?

Partly

Are sufficient details of methods and analysis provided to allow replication by others?

No

Reviewer Expertise:

Data science, deep learning, time series analysis, computer vision

I confirm that I have read this submission and believe that I have an appropriate level of expertise to state that I do not consider it to be of an acceptable scientific standard, for reasons outlined above.

# Response

**Published:** 2022-04-07

**Authors:** Wai Kit Wong

**Affiliations:** Multimedia University, Malaysia

**1. Reviewer: **The research contribution and novelty in terms of image processing are not well discussed in the manuscript. It is advised to discuss the methodology proposed by the authors to solve the problem statement.

**Author:**  The research contribution and novelty for this work is a new model of Malaysian banknotes counterfeit detection using watermarks image processing analysis and classifier with fuzzy logic. In particular, the three watermarks features: 1) see-thru windows, 2) Crescent and Star 3) Perfect see though register will be extracted from the one Ringgit and five Ringgit banknotes to determine the real/fake in a dynamic environment with ambiguous, distorted or imprecise banknotes images. Added in Second last paragraph of Section 1.

 A new Figure 4 is added with methodology proposed by the authors to solve the problem statement of banknote counterfeit detection. The detail of the algorithm is explained in Section 3.

**2. Reviewer :** Besides, it is very crucial to compare the performance of the proposed methodology with the state-of-the-art methods and evaluate its performance in terms of different error metrics.

**Author:** Comparison of the proposed banknote reader detection accuracy and processing speed is done with three state-of-the-art methods: 1) VGG16 model using 2D Convolution Layer (32 neural) at TensorFlow's Keras API [14], 2) MobileNet model using RMSprop Loss Function  (learning_rate=0.0001) at TensorFlow's Keras API [15] and 3) Fuzzy Logic Based Perceptual Image Hashing Algorithm [16].

 Experimental setup for method 1: Total of one hundred RM1 banknotes and one hundred RM5 banknotes are captured as the dataset for training the model. VGG16 model using 2D Convolution Layer (32 neural) at TensorFlow's Keras API being trained and tested with 100 real RM1, 100 real RM5, 100 fake RM1 and 100 fake RM5. The average time to load the model and build up the interpreter objects (Training time) was 60 seconds with batch size=32 and epochs=20 and the average inference time while modeling detecting banknote (Testing time) was 1 second. The test Accuracy was 60%.

 Experimental setup for method 2: It is understood that the model MobileNet with Loss Function RMSProp was Selected as best accuracy of about 96.80% in paper [15]. Convolutional Neural Networks using MobileNet model with Loss Function RMSProp (0.0001) optimization technique being trained with one hundred RM1 banknotes and one hundred RM5 banknotes and tested with 100 real RM1, 100 real RM5, 100 fake RM1 and 100 fake RM5. The average time to load the model and build up the interpreter objects (Training time) was 81 seconds with batch size=32 and epochs=20 and the average inference time while modeling detecting banknote (Testing time) was 1 second. The test Accuracy was 50%.

 Experimental setup for method 3: following paper [16] algorithm. Fuzzy Logic Based Perceptual Image Hashing Algorithm first sorting Database using Perceptual Hashing with one hundred RM1 banknotes and one hundred RM5 banknotes and tested with 100 real RM1, 100 real RM5, 100 fake RM1 and 100 fake RM5. The average time to load the model and build up the interpreter objects (test 100 banknotes) was 130 seconds and the average inference time while detecting banknote (Per banknote) was 1.30 seconds. The test Accuracy was 42%.

 The accuracy and required processing time for the experimented methods were summarized in Table 3. By comparing the above works on different Ringgit recognizers, it is observed that Fuzzy logic based light intensity variation watermark detection algorithm required longest processing time (both training and detection times for details watermark features extraction), however it has the best accuracy in detecting fake banknotes (minimum false positive and false negative cases) among the compared state-of-the-art methods. The VGG16 model, MobileNet model and Fuzzy Logic Based Perceptual Image Hashing Algorithm managed to be trained and detected the banknotes currency faster but with limitation of unable to accurately detecting fake banknotes (high false positive and false negative cases recorded) due to no watermarks detection consideration.  

 This write-up is added in Section 4 Experimental Session.

**3. Reviewer :** The author tried different IF-ELSE situations to detect the currency, however, the presentation of the same is very poor in the manuscript. It is advised to discuss these things in the form of block diagrams and Psuedo codes with proper formatting.

**Author:** The algorithm had been revised according to reviewers’ comments. IF-ELSE statement pseudocodes are well occupied in Section 3’s algorithm Step 4 onwards. General block diagram for the image processing algorithm well defined in Figure 4. Here in Section 3, the algorithm steps further details up the operation sequence of the banknote watermarks counterfeit detection.

**4. Reviewer:** The quality of figures in terms of resolution and aesthetics are very poor. It is advised to revise all figures with improved qualities.

**Author: **All figures revised with improved qualities.

**5. Reviewer :** The manuscript in the present form is like a project report, and not suitable for a research article. It is recommended to revise the manuscript with an improved case study that will discuss the research contributions in more detail than the hardware-software interface systems.

**Author:** More case studies were reviewed as per Reviewer 2 comment 20 as well. Whole Section 1 Introduction revised accordingly.
